# Determinants of lung function development from birth to age 5 years: an interrupted time series analysis of a South African birth cohort

**DOI:** 10.1016/S2352-4642(24)00072-5

**Published:** 2024-06

**Authors:** Carlyle McCready, Heather J Zar, Shaakira Chaya, Carvern Jacobs, Lesley Workman, Zoltan Hantos, Graham L Hall, Peter D Sly, Mark P Nicol, Dan J Stein, Anhar Ullah, Adnan Custovic, Francesca Little, Diane M Gray

**Affiliations:** a Department of Statistical Sciences, University of Cape Town, Cape Town, South Africa; b Department of Paediatrics and Child Health, University of Cape Town, Cape Town, South Africa; c South African Medical Research Council Unit on Child and Adolescent Health, University of Cape Town, Cape Town, South Africa; d Department of Psychiatry and Mental Health, University of Cape Town, Cape Town, South Africa; e South African Medical Research Council Unit on Risk & Resilience, University of Cape Town, Cape Town, South Africa; f Department of Anaesthesiology and Intensive Therapy, Semmelweis University, Budapest, Hungary; g Children's Lung Health, Telethon Kids Institute and School of Allied Health, Curtin University, Perth, WA, Australia; h Children's Health and Environment Program, Child Health Research Centre, University of Queensland, Brisbane, QLD, Australia; i Marshall Centre, School of Biomedical Sciences, University of Western Australia, Perth, WA, Australia; j National Heart and Lung Institute, Imperial College London, London, UK

## Abstract

**Background:**

Early life is a key period that determines long-term health. Lung development in childhood predicts lung function attained in adulthood and morbidity and mortality across the life course. We aimed to assess the effect of early-life lower respiratory tract infection (LRTI) and associated risk factors on lung development from birth to school age in a South African birth cohort.

**Methods:**

We prospectively followed children enrolled in a population-based cohort from birth (between March 5, 2012 and March 31, 2015) to age 5 years with annual lung function assessment. Data on multiple early-life exposures, including LRTI, were collected. The effect of early-life risk factors on lung function development from birth to age 5 years was assessed using the Generalised Additive Models for Location, Scale and Shape and Interrupted Time Series approach.

**Findings:**

966 children (475 [49·2%] female, 491 [50·8%] male) had lung function measured with oscillometry, tidal flow volume loops, and multiple breath washout. LRTI occurred in 484 (50·1%) children, with a median of 2·0 LRTI episodes (IQR 1·0–3·0) per child. LRTI was independently associated with altered lung function, as evidenced by lower compliance (0·959 [95% CI 0·941–0·978]), higher resistance (1·028 [1·016–1·041]), and higher respiratory rate (1·018 [1·063–1·029]) over 5 years. Additional impact on lung function parameters occurred with each subsequent LRTI. Respiratory syncytial virus (RSV) LRTI was associated with lower expiratory flow ratio (0·97 [0·95–0·99]) compared with non-RSV LRTI. Maternal factors including allergy, smoking, and HIV infection were also associated with altered lung development, as was preterm birth, low birthweight, female sex, and coming from a less wealthy household.

**Interpretation:**

Public health interventions targeting LRTI prevention, with RSV a priority, are vital, particularly in low-income and middle-income settings.

**Funding:**

UK Medical Research Council Grant, The Wellcome Trust, The Bill & Melinda Gates Foundation, US National Institutes of Health Human Heredity and Health in Africa, South African Medical Research Council, Hungarian Scientific Research Fund, and European Respiratory Society.

## Introduction

Early-life events are key determinants of long-term health, with harmful exposures setting a trajectory for chronic illness or mortality throughout the life course.^1^ Diminished lung function at the physiological peak in early adulthood is associated with subsequent adverse health outcomes into middle and late adulthood, including higher risk of chronic obstructive pulmonary disease, cardiovascular and cerebrovascular events, sudden cardiac death, and premature death from all causes.^2^, ^3^, ^4^ Impaired lung function in childhood is associated with low lung function in adulthood.^5^ Many antenatal and early-life factors are associated with diminished lung growth and development throughout childhood. For example, lower respiratory tract infection (LRTI) in early childhood has been associated with impairments in lung function throughout the life course^6^ and premature mortality,^7^ but most studies have been done in high-income countries (HICs).^6^, ^8^ For example, the Tucson Children's study, a US birth cohort, showed pneumonia in the first 3 years of life to be associated with reduced spirometry in childhood that persisted into early adulthood.^9^ However, most children globally live in low-income and middle-income countries (LMICs) and are particularly at risk of impairment in lung function given the high burden of early-life risk factors, especially high incidence of severe LRTI,^10^ exposure to polluted air, and social disadvantage. Identifying which factors impair healthy lung development during early life in LMICs is important to specify targets for strengthening health across the life course.

There are comparatively few data on the development of lung function during pre-school age compared with school age and adolescence, in part because of the difficulties in measuring lung function in early childhood. Technological advances have allowed non-invasive measurements of lung function soon after birth, during early childhood, and in adulthood, allowing the investigation of lung function development in relation to early-life events and exposures. These measurements are possible even in community settings^11^ in lower resourced communities where children are particularly at risk for poor respiratory health. In the Drakenstein Child Health Study (DCHS), a South African birth cohort, we reported that LRTI in early life was associated with impairment in lung function at age 2 years, with additional loss in lung function for each recurrent episode, independent of risk inferred by baseline lung function.^12^, ^13^ This effect on lung function can occur through various mechanisms, with variable contributions from additional factors including the infectious organism, severity, and recurrence.^13^ Other risk factors such as environmental exposures (eg, air pollution and tobacco smoke),^14^ immunological factors (eg, HIV exposure and atopy),^15^ maternal health (eg, atopy and psychological stress),^16^ and social deprivation (eg, overcrowding, food insecurity, and poor maternal education) can further affect healthy lung development. In the current study, we aimed to assess the effect of early-life LRTI and associated early-life risk factors on lung function development from birth to age 5 years in the DCHS.

**Research in context**

**Evidence before this study**

Early-life events are key determinants of long-term health, with harmful exposures setting a trajectory for chronic illness or premature mortality throughout the life course. We searched PubMed, Google Scholar, and EMBASE; journal reference lists; and conference abstracts from database inception to Oct 30, 2023, for longitudinal birth cohort studies (birth cohort; lung function*; infant and/or child*; antenatal and/or pregnancy exposure) assessing the effect of early-life exposures on lung function from birth to school age. Few longitudinal cohort studies included baseline lung function and lung function throughout preschool years. Longitudinal birth cohort studies are mostly from high-income countries (HICs) and show that lung function reached at school age is predictive of the adult physiological peak. Further, lung function attained at adulthood predicts adverse health outcomes, including chronic respiratory disease, cardiovascular or cerebrovascular events, and mortality. Antenatal and early-life risk factors, including childhood lower respiratory tract infections (LRTIs), are identified risk factors for impairment of lung function development in HICs. Data from low-income and middle-income countries (LMICs) remain rare, along with data on the effect of baseline lung function and the timing and nature of lung function damage in early life, a critical time of development.

**Added value of this study**

This study of a South African birth cohort comprehensively assessed lung function measures at multiple timepoints from birth throughout early childhood in a LMIC context with high LRTI incidence. The effect of key exposures on healthy lung development independent of the risk conferred by baseline lung function was identified, including LRTI. Importantly, early-life LRTI, even ambulatory disease, negatively affected lung development, with a more marked effect with severe infection and each subsequent event. Respiratory syncytial virus (RSV) LRTI was associated with decreased expiratory flow ratios up to age 5 years, consistent with developing obstructive lung disease. The effect on healthy lung development is worst in those with exposure to multiple risk factors.

**Implications of the available evidence**

The results identify important targets for intervention, including LRTI and RSV LRTI prevention; maternal health; and socioeconomic disadvantage, particularly in LMICs. Future research needs to focus on the clinical implications of impaired early-life lung function. In addition, identifying subgroups of children who catch-up or worsen overtime will be important in optimising prevention and management strategies to strengthen lifelong health.

## Methods

### Study design and participants

The DCHS is a population-based birth cohort (appendix pp 3–4), as described previously.^17^ Pregnant women were enrolled during their second trimester at two public health clinics in a peri-urban area outside Cape Town, South Africa, between 2012 and 2015. All births occurred at the single public hospital. The study was approved by the Human Research Ethics Committee of the Faculty of Health Sciences, University of Cape Town, South Africa (HREC 401/2009, 423/2012). Mothers gave written informed consent at enrolment and were re-consented annually.

### Procedures

Follow-up visits were performed at 6, 10, and 14 weeks, at 6, 9, and 12 months, and every 6 months thereafter until age 5 years. Visits included clinical examination and interviews by trained staff to collect data on environmental exposures, household characteristics, maternal factors, nutrition, and symptoms. Details are summarised in the appendix (p 5). Maternal depression was measured using the Edinburgh Postnatal Depression Scale. Preterm was defined as being born before 37 weeks gestational age, very preterm as before 32 weeks, and moderate-to-late preterm as 32 to less than 37 weeks. Wheeze phenotypes (never, transient, late-onset, and recurrent wheezing) were previously derived and reported.^18^

Active surveillance for LRTI was done through community health workers, use of mobile phones, a dedicated study contact person available 24 h a day, and a network of study staff at community-based sites. Each suspected case of LRTI was examined by study staff to confirm the presence of LRTI.^18^ LRTI episodes were defined as non-severe and severe by WHO classification of childhood pneumonia.^19^ At each LRTI or wheezing episode, a nasopharyngeal swab was obtained for multiplex real-time PCR with FTDResp33 (Fast-track Diagnostics, Eschsur-Alzet, Luxembourg) to identify up to 33 organisms, including respiratory syncytial virus (RSV) and rhinovirus.^13^, ^20^

Lung function was assessed at 6 weeks, 1 year, and annually until age 5 years. The 5-year test was collected up to but not including the sixth birthday. Comprehensive lung function measurements were collected (appendix p 6). 21, 22, 23 Tidal breathing flow volume loops were used to measure respiratory rate, tidal volume, and expiratory flow ratio time to peak tidal expiratory flow by total expiratory time (t_PTEF_/t_E_). An increased respiratory rate and low tidal volume or t_PTEF_/t_E_ would reflect lower lung function and a reduced t_PTEF_/t_E_ is associated with airflow obstruction. Multiple breath washout (MBW) was used to measure functional residual capacity (FRC) and lung clearance index (LCI), a measure of ventilation homogeneity. A high LCI reflects impairment, and changes in FRC could reflect either poor lung growth if low, or air trapping of obstructive lung disease if high. Respiratory impedance was measured by oscillometry, expressed as respiratory system resistance and reactance (appendix p 6). Oscillometry included both conventional (multiple frequency) and intrabreath (single frequency) methods.^22^ From the multiple-frequency respiratory impedance data, the parameters of resistance and compliance were estimated by model fitting; from intrabreath measurements, end-expiratory resistance (R_ee_) and end-expiratory reactance (X_ee_) were determined. A high respiratory system resistance and a low respiratory system reactance or compliance indicates poorer lung function, manifesting as stiffer lungs, that may be due to airway disease with obstruction or lung parenchymal involvement. In participants aged 6 weeks to 2 years, oscillometry was performed during unsedated quiet sleep via a filter and a face mask. From age 3 years onwards, oscillometry was done awake with children seated. Lung function was deferred until at least 4 weeks after an LRTI or wheezing episode.

### Statistical analysis

We investigated lung function development throughout early childhood using the Generalised Additive Models for Location, Scale and Shape (GAMLSS) approach in combination with an Interrupted Time Series (ITS) approach. This approach allowed us to model changes in lung function development over time, accounting for the effect of a step-change that occurred when testing changed from asleep to awake measurements at age 3 years. We modelled FRC, LCI, tidal volume, respiratory rate, t_PTEF_/t_E_, resistance, compliance, R_ee_, and X_ee_ from birth to age 5 years. To account for the effect of body size on lung function, we adjusted the lung function measurements for height (as a proxy for body size) using a multiplicative model approach (appendix p 7).

We used GAMLSS models to investigate early-life associations with lung function development. The effect of LRTI was further explored for timing, frequency, hospitalisation, and causal organism (RSV or rhinovirus). The effect of maternal HIV exposure was further explored by maternal HIV disease severity. Models were developed using a three parameter (μ, σ, and ν) Box-Cox Cole and Green (BCCG) distribution and a four parameter (μ, σ, ν, and τ) Box-Cox t distribution with increasing complexity (appendix p 8). The best fitting model for each outcome was selected using the Generalised Akaike information criterion. A minimum set of confounders was identified using a directed acyclic graph (appendix p 9).

The X_ee_ outcome was inverted, and a constant was added to model the outcome using a BCCG distribution under the GAMLSS framework. Lung function outcomes were modelled using a log link for the *μ* parameter. Results are reported as exponentiated coefficients for *μ* on the log scale with 95% CIs. Adjusted p values for multiple comparisons were calculated using the Benjamini and Hochberg method.^24^ Analyses were conducted in R version 3.6.3.

### Role of the funding source

The funders of the study had no role in study design, data collection, data analysis, data interpretation, or writing of the report.

## Results

**Figure 1:**
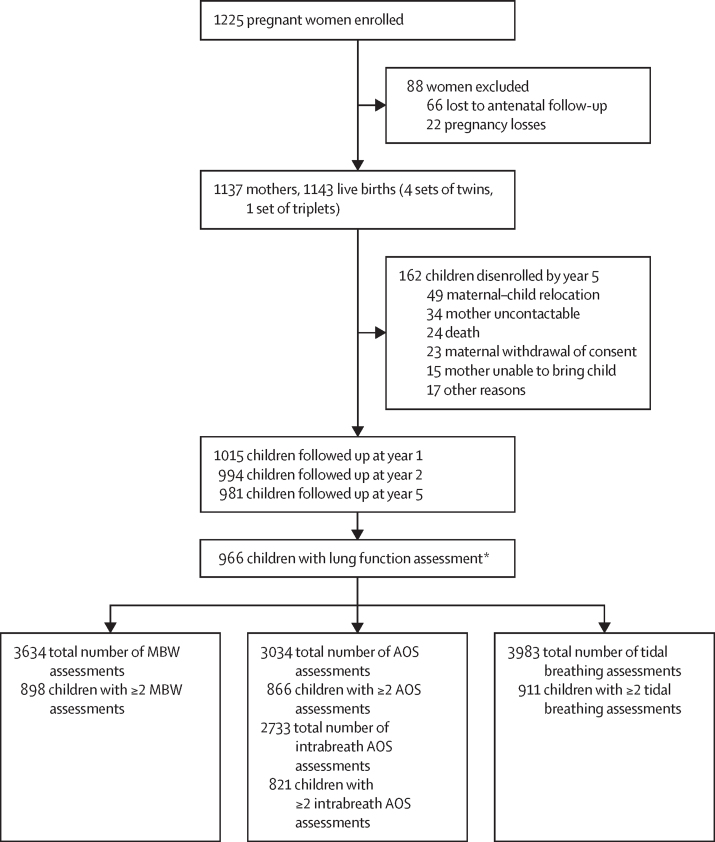
Trial profile AOS=airwave oscillometry. MBW=multiple breath washout. *632 (62·3%) of 1015 children at year 1, 639 (64·3%) of 994 at year 2, and 825 (84·1%) of 981 at year 5 with lung function included in the analysis.

1137 mothers (median age 25·8 years [IQR 22·0–30·8]) were enrolled between March 5, 2012, and March 31, 2015, with 1143 livebirths (figure 1). Retention and lung function testing success were high in this cohort, with 923 (80·8%) children having 2 or more lung function measurements between birth and age 5 years. This analysis included 966 (84·5%) children who had at least one lung function measurement across six timepoints from age 6 weeks to 5 years. Excluded children had similar characteristics to those included; however, the excluded group had higher socioeconomic status, lower prevalence of antenatal smoking, higher frequency of preterm birth, and younger maternal age (appendix p 10).

Characteristics of the child study population (475 [49·2%] female, 491 [50·8%] male) are described in table 1. The cohort is of Black South African (Black African 518 [53·6%]) or mixed (448 [46·4%]) ancestry by self-identification and primarily of low socioeconomic status, with 845 (87·5%) households earning less than R5000 (<US$367) per month. Prevalence of postnatal maternal smoking was high. Postnatally, more than a third of mothers reported intimate partner violence, and depression was common. 150 (15·5%) children were born preterm, most of whom were late preterm. 211 (21·8%) children were HIV exposed uninfected (HEU), but only two (<1·0%) children were living with HIV.

**Table 1:** Participant characteristics

		Participants*
**Child characteristics**		
Sex†		
	Female	475 (49·2%)
	Male	491 (50·8%)
Preterm (<37 weeks gestational age)		150 (15·5%)
Moderate to late preterm (32 to <37 weeks gestational age)		104 (10·8%)
HIV exposed uninfected		211 (21·8%)
Children living with HIV		2 (0·2%)
Birthweight Z score		−0·25 (−0·96 to 0·41)
**LRTI in first year**		
Number of children with ≥1 LRTI episodes before 12 months		366 (37·9%)
Number of LRTI episodes before 12 months		593
Hospitalised LRTI (number of children) before 12 months		128 (13·3%)
Hospitalised LRTI (number of episodes) before 12 months		161
RSV LRTI (number of children) before 12 months		119 (12·3%)
RSV LRTI (number of episodes) before 12 months		123
Hospitalised RSV LRTI (number of children) before 12 months		45 (4·7%)
Hospitalised RSV LRTI (number of episodes) before 12 months		46
Rhinovirus LRTI (number of children) before 12 months		119 (12·3%)
Rhinovirus LRTI (number of episodes) before 12 months		148
Hospitalised rhinovirus-LRTI (number of children) before 12 months		18 (1·9%)
Hospitalised rhinovirus-LRTI (number of episodes) before 12 months		20
**LRTI**		
LRTI (number of children)		484 (50·1%)
LRTI (number of episodes)		1053
Hospitalised LRTI (number of children)		162 (16·8%)
Hospitalised LRTI (number of episodes)		226
RSV LRTI (number of children)		165 (17·1%)
RSV LRTI (number of episodes)		180
Hospitalised RSV LRTI (number of children)		55 (5·7%)
Hospitalised RSV LRTI (number of episodes)		56
Rhinovirus LRTI (number of children)		203 (21·0%)
Rhinovirus LRTI (number of episodes)		311
Hospitalised rhinovirus LRTI (number of children)		33 (3·4%)
Hospitalised rhinovirus LRTI (number of episodes)		42
**Wheeze phenotypes**		
Never wheeze		472/939 (50·3%)
Early transient		215/939 (22·9%)
Late onset		103/939 (11·0%)
Recurrent		149/939 (15·9%)
**Maternal characteristics**		
Age at enrolment (years)		26·1 (22·1 to 30·8)
Postnatal smoking		311/961 (32·4%)
Maternal self-reported asthma or allergy		66 (6·8%)
Postnatal intimate partner violence		357/913 (39·1%)
Maternal depression		230/907 (25·4%)
Maternal education		
	Completed primary	71 (7·4%)
	Any secondary	535 (55·4%)
	Completed secondary	305 (31·6%)
	Any tertiary	55 (5·7%)
**Socioeconomic status**		
Household income (per month)		
	<R1000 (US$ 67)	338/957 (35·3%)
	R1000–5000 (US$ 67–336)	507/957 (53·0%)
	>R5000 (US$ 336)	112/957 (11·7%)
Ranges for household size quartiles (number of people) in this cohort		
	Small	1–3
	Small to medium	4
	Medium to large	5–6
	Large	7–18
Ranges for asset ownership quartiles in this cohort		
	Low	1–5
	Low to medium	6–7
	Medium to high	8
	High	9–11

Data shown are n (%), median (IQR), n, or n/N (%). LRTI=lower respiratory tract infection. RSV=respiratory syncytial virus.

* 966 children with at least one lung function measurement for all variables, unless shown.

† Determined by clinical examination at birth.

LRTI was common. During the 4830 child-years of follow-up, 1053 LRTI episodes occurred, a median of 2·0 (IQR 1·0–3·0) episodes per child (table 1).

There were 13 384 lung function measurements available: 3983 tidal breathing, 3634 MBW, 3034 conventional oscillometry, and 2733 with intrabreath oscillometry measurements (appendix pp 11–12). The development of lung function parameters from birth to age 5 years by sex for each measure is presented in the appendix (pp 13–16), as is function tracked from birth to age 5 years (appendix pp 17–18). The effect of age and exposure to various risk factors on the development of lung function parameters, including p values adjusted for multiple testing, is shown (Table 2, Table 3, appendix pp 19–20).

**Table 2:** Association of early life risk factors with the development of multiple breath washout and tidal breathing parameters from birth to age 5 years; multivariate model

			FRC*		LCI*		Respiratory rate*		**Tidal volume**†		**t**_PTEF_**/t**_E_*	
			Effect size (95% CI)	p value (p value adj)	Effect size (95% CI)	p value (p value adj)	Effect size (95% CI)	p value (p value adj)	Effect size (95% CI)	p value (p value adj)	Effect size (95% CI)	p value (p value adj)
Age (months) for children aged 0–3 years†			1·024 (1·023–1·025)	<0·0001 (<0·0001)	0·997 (0·997–0·997)	<0·0001 (<0·0001)	0·983 (0·983–0·984)	<0·0001 (<0·0001)	1·025 (1·025–1·026)	<0·0001 (<0·0001)	0·985 (0·984–0·987)	<0·0001 (<0·0001)
Adjustment for change in lung function measurement technique at age 3 years			2·133 (2·063–2·205)	<0·0001 (<0·0001)	1·004 (0·984–1·024)	0·68 (0·91)	0·720 (0·695–0·745)	<0·0001 (<0·0001)	2·324 (2·261–2·389)	<0·0001 (<0·0001)	1·312 (1·249–1·377)	<0·0001 (<0·0001)
Combined effect of age 0-3 years and adjustment for change in lung function measurement technique			0·980 (0·979–0·981)	<0·0001 (<0·0001)	1·002 (1·002–1·003)	<0·0001 (<0·0001)	1·017 (1·016–1·018)	<0·0001 (<0·0001)	0·976 (0·975–0·977)	<0·0001 (<0·0001)	1·011 (1·009–1·012)	<0·0001 (<0·0001)
Child characteristics												
	Male *vs* female		1·062 (1·051–1·073)	<0·0001 (<0·0001)	0·998 (0·993–1·003)	0·39 (0·87)	0·999 (0·989–1·010)	0·98 (0·98)	1·032 (1·023–1·041)	<0·0001 (<0·0001)	0·991 (0·975–1·007)	0·25 (0·37)
	Preterm (<37 weeks) *vs* ≥37 weeks		0·982 (0·968–0·997)	0·016 (0·050)	0·999 (0·993–1·006)	0·87 (0·97)	1·015 (1·0005–1·029)	0·042 (0·062)	0·975 (0·963–0·987)	<0·0001 (<0·0001)	0·965 (0·944–0·986)	0·0020 (0·0060)
	HIV exposed uninfected *vs* HIV unexposed		1·0006 (0·987–1·014)	0·93 (0·94)	1·008 (1·002–1·014)	0·016 (0·10)	1·005 (0·991–1·018)	0·49 (0·98)	1·022 (1·011–1·034)	<0·0001 (<0·0001)	0·996 (0·976–1·017)	0·74 (0·80)
	Birthweight Z score		1·006 (1·001–1·011)	0·018 (0·26)	1·0009 (0·998–1·003)	0·42 (0·86)	0·993 (0·988–0·998)	0·0064 (0·016)	1·013 (1·009–1·017)	<0·0001 (<0·0001)	1·007 (0·999–1·015)	0·052 (0·10)
Wheeze phenotype												
	Early transient *vs* never		0·994 (0·981–1·008)	0·41 (0·51)	1·009 (1·003–1·016)	0·0043 (0·036)	1·005 (0·992–1·019)	0·44 (0·53)	0·992 (0·981–1·003)	0·17 (0·30)	1·017 (0·996–1·038)	0·11 (0·19)
	Late onset *vs* never		1·017 (0·999–1·034)	0·062 (0·13)	0·995 (0·987–1·004)	0·28 (0·81)	0·978 (0·961–0·995)	0·012 (0·027)	0·992 (0·978–1·007)	0·31 (0·48)	1·011 (0·984–1·038)	0·42 (0·55)
	Recurrent *vs* never		1·012 (0·996–1·028)	0·15 (0·25)	1·008 (1·001–1·015)	0·047 (0·26)	0·980 (0·964–0·996)	0·014 (0·029)	1·006 (0·992–1·019)	0·41 (0·54)	0·960 (0·936–0·984)	0·0020 (0·0060)
	LRTI (ever) *vs* never		0·987 (0·975–0·998)	0·022 (0·055)	0·999 (0·994–1·005)	0·91 (0·97)	1·018 (1·0063–1·029)	0·0024 (0·0067)	0·993 (0·984–1·003)	0·18 (0·31)	1·002 (0·984–1·020)	0·79 (0·82)
Maternal characteristics												
	Postnatal intimate partner violence *vs* none		1·015 (1·005–1·026)	0·0051 (0·021)	0·997 (0·992–1·002)	0·29 (0·81)	1·005 (0·994–1·015)	0·37 (0·49)	1·001 (0·991–1·009)	0·98 (0·98)	0·982 (0·966–0·998)	0·033 (0·075)
	Maternal asthma or allergy *vs* none		1·016 (0·996–1·036)	0·11 (0·21)	0·998 (0·988–1·007)	0·67 (0·91)	1·021 (1·002–1·041)	0·034 (0·059)	0·986 (0·969–1·002)	0·086 (0·18)	1·030 (0·999–1·061)	0·053 (0·10)
	Postnatal smoking *vs* none		0·984 (0·973–0·996)	0·0092 (0·032)	1·0002 (0·995–1·006)	0·93 (0·97)	0·979 (0·968–0·990)	<0·0001 (<0·0001)	1·008 (0·999–1·018)	0·084 (0·18)	0·976 (0·959–0·994)	0·0084 (0·021)
Maternal education												
	Secondary *vs* primary		0·986 (0·966–1·005)	0·16 (0·25)	0·997 (0·988–1·006)	0·54 (0·90)	1·023 (1·003–1·044)	0·022 (0·042)	1·012 (0·995–1·029)	0·15 (0·29)	1·003 (0·973–1·034)	0·83 (0·83)
	Completed secondary *vs* primary		0·995 (0·974–1·016)	0·64 (0·71)	0·997 (0·987–1·007)	0·58 (0·91)	1·020 (0·998–1·042)	0·068 (0·094)	1·007 (0·989–1·025)	0·43 (0·54)	1·014 (0·982–1·047)	0·30 (0·54)
	Any tertiary *vs* primary		0·979 (0·951–1·008)	0·15 (0·25)	0·999 (0·986–1·013)	0·97 (0·97)	0·999 (0·971–1·029)	0·98 (0·98)	1·052 (1·026–1·077)	<0·0001 (<0·0001)	0·970 (0·927–1·015)	0·19 (0·29)
Socioeconomic status												
	Asset ownership											
		Low to medium *vs* low	1·003 (0·988–1·018)	0·66 (0·72)	1·001 (0·994–1·008)	0·73 (0·91)	0·984 (0·969–0·999)	0·038 (0·059)	1·0006 (0·988–1·013)	0·92 (0·96)	0·994 (0·971–1·017)	0·61 (0·73)
		Medium to high *vs* low	1·0006 (0·985–1·016)	0·94 (0·94)	1·003 (0·995–1·009)	0·48 (0·86)	0·972 (0·957–0·987)	<0·0001 (<0·0001)	1·005 (0·991–1·017)	0·48 (0·54)	0·955 (0·932–0·978)	<0·0001 (<0·0001)
		High *vs* low	1·018 (1·001–1·035)	0·033 (0·075)	0·995 (0·987–1·002)	0·18 (0·64)	0·954 (0·939–0·970)	<0·0001 (<0·0001)	1·022 (1·008–1·036)	0·0020 (0·0051)	0·963 (0·938–0·988)	0·0041 (0·0050)
	Household size											
		Small to medium (4) *vs* small (1–3)	0·993 (0·977–1·008)	0·34 (45)	1·005 (0·998–1·012)	0·18 (0·64)	0·990 (0·975–1·005)	0·22 (0·31)	1·011 (0·997–1·024)	0·11 (0·20)	1·004 (0·981–1·028)	0·71 (0·77)
		Medium to large (5–6) *vs* small (1–3)	1·028 (1·012–1·045)	<0·0001 (<0·0001)	1·0002 (0·993–1·008)	0·95 (0·97)	0·989 (0·974–1·005)	0·21 (0·31)	1·019 (1·005–1·033)	0·0050 (0·013)	1·017 (0·992–1·042)	0·17 (0·27)
		Large (7–18) *vs* small (1–3)	1·005 (0·989–1·021)	0·55 (0·65)	1·002 (0·994–1·009)	0·61 (0·94)	0·989 (0·974–1·005)	0·18 (0·28)	0·998 (0·985–1·012)	0·79 (0·86)	1·029 (1·004–1·055)	0·021 (0·058)
	Household income (per month)											
		R1000–5000 (US$67–336) *vs* <R1000 (US$67)	1·012 (1·001–1·024)	0·032 (0·068)	0·999 (0·994–1·005)	0·86 (0·97)	1·004 (0·993–1·015)	0·43 (0·52)	1·004 (0·994–1·013)	0·44 (0·54)	0·979 (0·962–0·996)	0·015 (0·25)
		>R5000 (US$336) *vs* <R1000 (US$67)	1·021 (1·003–1·039)	0·019 (0·052)	0·998 (0·989–1·005)	0·43 (0·86)	1·002 (0·984–1·019)	0·85 (0·92)	0·993 (0·979–1·007)	0·33 (0·48)	0·995 (0·969–1·022)	0·73 (0·80)

All models include a child-specific random effect, all variables in the table are included in model. All lung function measurements except LCI and t_PTEF_/t_E_ were standardised for height at test. Adjusted p values were adjusted for multiple comparisons, an approach by Benjamini and Hochberg.^24^ Effect size is the exponent of the μ coefficients (percentage change of lung function measure associated with the predictor variable in question compared to the reference category). FRC=functional residual capacity. LCI=lung clearance index. t_PTEF_/t_E_=time to peak tidal expiratory time over total expiratory time. LRTI=lower respiratory tract infection.

* Box-Cox t distribution.

† Box-Cox Cole and Green distribution. † The effect is per 1-month increase in age.

**Table 3:** Association of early life risk factors with the development of oscillometry parameters from birth to age 5 years; multivariate model

			Resistance*		Compliance*		**R**_ee_*		**Inversed X**_ee_†	
			Effect size (95% CI)	p value (p value adj)	Effect size (95% CI)	p value (p value adj)	Effect size (95% CI)	p value (p value adj)	Effect size (95% CI)	p value (p value adj)
Age (months) for children aged 0–3 years‡			0·996 (0·995–0·998)	p<0·0001 (p<0·0001)	1·008 (1·006–1·009)	p<0·0001 (p<0·0001)	0·987 (0·986–0·987)	p<0·0001 (p<0·0001)	0·987 (0·986–0·989)	p<0·0001 (p<0·0001)
Adjustment for change in lung function measurement technique at age 3 years			0·533 (0·515–0·552)	p<0·0001 (p<0·0001)	2·126 (2·014–2·245)	p<0·0001 (p<0·0001)	0·621 (0·596–0·647)	p<0·0001 (p<0·0001)	0·618 (0·593–0·644)	(p<0·0001) (p<0·0001)
Combined effect of age 0-3 years and adjustment for change in lung function measurement technique			1·001 (0·999–1·003)	0·14 (0·29)	0·995 (0·993–0·997)	p<0·0001 (p<0·0001)	1·009 (1·007–1·010)	p<0·0001 (p<0·0001)	1·009 (1·007–1·010)	(p<0·0001) (p<0·0001)
Child characteristics										
	Male *vs* female		0·978 (0·967–0·989)	p<0·0001 (p<0·0001)	1·051 (1·032–1·069)	p<0·0001 (p<0·0001)	0·984 (0·970–0·998)	p<0·0001 (p<0·0001)	0·984 (0·970–0·998)	0·033 (0·75)
	Preterm (<37 weeks) *vs* ≥37 weeks		1·059 (1·042–1·075)	p<0·0001 (p<0·0001)	0·916 (0·894–0·940)	p<0·0001 (p<0·0001)	1·062 (1·041–1·084)	p<0·0001 (p<0·0001)	1·064 (1·042–1·086)	p<0·0001 (p<0·0001)
	HIV exposed uninfected *vs* HIV unexposed		1·011 (0·996–1·026)	0·14 (0·29)	1·003 (0·979–1·027)	0·81 (0·81)	0·998 (0·98–1·018)	0·88 (0·092)	1·004 (0·998–1·009)	0·89 (0·94)
	Birthweight Z score		0·993 (0·987–0·998)	0·011 (0·034)	1·021 (1·013–1·030)	p<0·0001 (p<0·0001)	0·995 (0·988–1·002)	0·15 (0·25)	0·994 (0·987–1·001)	0·12 (0·20)
Wheezing phenotype										
	Early transient *vs* never		0·994 (0·979–1·008)	0·38 (0·56)	1·013 (0·989–1·037)	0·27 (0·41)	1·008 (0·989–1·026)	0·41 (0·57)	1·009 (0·991–1·029)	0·31 (0·45)
	Late onset *vs* never		1·026 (1·007–1·045)	0·0068 (0·024)	0·972 (0·943–1·002)	0·065 (0·13)	1·045 (1·021–1·070)	p<0·0001 (p<0·0001)	1·048 (1·022–1·073)	0·0020 (0·0071)
	Recurrent *vs* never		1·009 (0·992–1·027)	0·28 (0·53)	0·948 (0·922–0·975)	p<0·0001 (p<0·0001)	1·039 (1·016–1·063)	p<0·0001 (p<0·0001)	1·041 (1·017–1·065)	p<0·0001 (p<0·0001)
	LRTI (ever) *vs* never		1·028 (1·016–1·041)	p<0·0001 (p<0·0001)	0·959 (0·941–0·978)	p<0·0001 (p<0·0001)	1·034 (1·017–1·051)	p<0·0001 (p<0·0001)	1·032 (1·015–1·049)	p<0·0001 (p<0·0001)
Maternal characteristics										
	Postnatal intimate partner violence *vs* none		0·991 (0·979–1·003)	0·13 (0·29)	1·013 (0·994–1·032)	0·18 (0·32)	0·983 (0·968–0·997)	0·019 (0·047)	0·982 (0·967–0·997)	0·021 (0·050)
	Maternal asthma or allergy *vs* none		0·995 (0·974–1·016)	0·65 (0·77)	1·017 (0·983–1·053)	0·32 (0·41)	0·964 (0·938–0·991)	0·0091 (0·025)	0·966 (0·938–0·993)	0·016 (0·050)
	Postnatal smoking *vs* none		1·029 (1·016–1·042)	p<0·0001 (p<0·0001)	1·003 (0·984–1·023)	0·74 (0·80)	1·018 (1·002–1·034)	0·024 (0·054)	1·019 (1·003–1·036)	0·019 (0·050)
Maternal education										
	Secondary *vs* primary		1·010 (0·989–1·032)	0·34 (0·56)	0·982 (0·949–1·016)	0·31 (0·41)	1·001 (0·975–1·028)	0·92 (0·92)	1·001 (0·974–1·029)	0·94 (0·94)
	Completed secondary *vs* primary		1·027 (1·004–1·049)	0·022 (0·061)	0·984 (0·949–1·021)	0·41 (0·46)	1·024 (0·995–1·054)	0·11 (0·21)	1·024 (0·994–1·054)	0·12 (0·20)
	Any tertiary *vs* primary		0·996 (0·965–1·029)	0·85 (0·88)	0·969 (0·921–1·021)	0·24 (0·40)	0·997 (0·957–1·039)	0·91 (0·92)	0·999 (0·957–1·04)	0·91 (0·94)
Socioeconomic status										
	Asset ownership									
		Low to medium *vs* low	1·0008 (0·985–1·017)	0·92 (0·92)	1·012 (0·986–1·038)	0·37 (0·44)	1·012 (0·991–1·033)	0·27 (0·40)	1·010 (0·989–1·032)	0·35 (0·49)
		Medium to high *vs* low	0·997 (0·989–1·015)	0·77 (0·85)	1·034 (1·006–1·063)	0·015 (0·037)	1·003 (0·981–1·024)	0·81 (0·92)	1·002 (0·979–1·024)	0·88 (0·94)
		High *vs* low	1·005 (0·987–1·024)	0·56 (0·77)	0·986 (0·958–1·015)	0·33 (0·41)	1·002 (0·979–1·025)	0·89 (0·92)	1·001 (0·978–1·025)	0·91 (0·94)
	Household size									
		Small to medium (4) *vs* small (1–3)	1·007 (0·991–1·024)	0·38 (0·56)	1·018 (0·992–1·046)	0·18 (0·32)	0·985 (0·965–1·006)	0·17 (0·26)	0·983 (0·962–1·005)	0·13 (0·20)
		Medium to large (5–6) *vs* small (1–3)	1·008 (0·991–1·026)	0·35 (0·56)	1·004 (0·976–1·032)	0·79 (0·81)	0·992 (0·970–1·014)	0·47 (0·61)	0·989 (0·967–1·013)	0·38 (0·50)
		Large (7–18) *vs* small (1–3)	0·995 (0·978–1·013)	0·59 (0·77)	1·046 (1·017–1·075)	0·0020 (0·0050)	0·979 (0·957–1·001)	0·056 (0·12)	0·977 (0·956–1·000)	0·049 (0·10)
	Household income (per month)									
		R1000–5000 (US$67–336) *vs* <R1000 (US$67)	1·003 (0·991–1·015)	0·63 (0·77)	1·010 (0·991–1·029)	0·31 (0·41)	1·004 (0·988–1·019)	0·64 (0·80)	1·002 (0·986–1·018)	0·81 (0·94)
		>R5000 (US$336) *vs* <R1000 (US$67)	0·997 (0·979–1·016)	0·78 (0·85)	1·029 (0·999–1·061)	0·057 (0·13)	0·982 (0·958–1·005)	0·13 (0·23)	0·979 (0·955–1·004)	0·093 (0·18)

All models include a child-specific random effect, all variables in table included in the model. P values were adjusted for multiple comparisons, an approach by Benjamini and Hochberg.^24^ Effect size is the exponent of the μ coefficients (percentage change of lung function measure associated with thepredictor variable in question compared to the reference category). IPV=intimatepartner violence. LRTI=lower respiratory tract infection. R_ee_=respiratory resistance at the end of expiration. X_ee_=respiratory reactance at the end of expiration.

* Box-Cox t distribution.

† Box-Cox Cole and Green distribution.

‡ The effect is per 1-month increase in age.

After adjusting for the possible confounders, including birthweight Z score and height at test, male children had larger lung volumes than female children up to age 5 years, reflecting the increased lung size and early airway dysynapsis (larger airway to lung ratio) in males. There was a step change in most measures with a change in testing technique at age 3 years. Changes in lung function were most marked during early life, with a decreased rate of change per month after age 3 years (appendix pp 11–12).

Children born preterm had lower size-adjusted lung volumes (FRC and tidal volume), compliance, and t_PTEF_/t_E,_ up to age 5 years compared with term children. These results probably reflect both early impaired lung development and an increased vulnerability to lung damage from additional early-life harm. Children who were HEU had mildly increased LCI compared with HIV-unexposed children. The altered lung function in children who were HEU was associated with maternal HIV disease severity, with the increased LCI noted only in infants whose mothers had a detectable HIV viral load during pregnancy (appendix p 21). Higher birthweight Z score was associated with better lung function, larger height-adjusted volumes, higher expiratory flow ratio and compliance, and lower respiratory rate and resistance—all consistent with sustained good lung and airway growth throughout childhood. Maternal asthma and allergy were associated with higher respiratory rate and lower tidal volume over 5 years.

Maternal smoking was associated with higher resistance and decreased t_PTEF_/t_E_ and respiratory rate, suggestive of airway disease. Postnatal intimate partner violence was associated with lower t_PTEF_/t_E_ and R_ee_ and higher FRC.

All socioeconomic status measures were associated with aspects of altered lung function development. Compared with maternal primary education, maternal tertiary education was associated with higher tidal volume and maternal secondary education was associated with higher resistance. Compared with low asset ownership, high asset ownership was associated with increased tidal volume, lower respiratory rate, and lower t_PTEF_/t_E_. Larger household size was associated with higher compliance, FRC, tidal volume, and t_PTEF_/t_E_. Children from higher income households had higher FRC than those from lower income households.

Compared with children in the never wheeze cluster, early transient wheeze was associated with increased LCI. Late-onset wheeze was associated with higher resistance and lower respiratory rate. Recurrent wheeze was associated with higher R_ee_ and LCI and lower compliance, t_PTEF_/t_E_, and respiratory rate.

**Figure 2:**
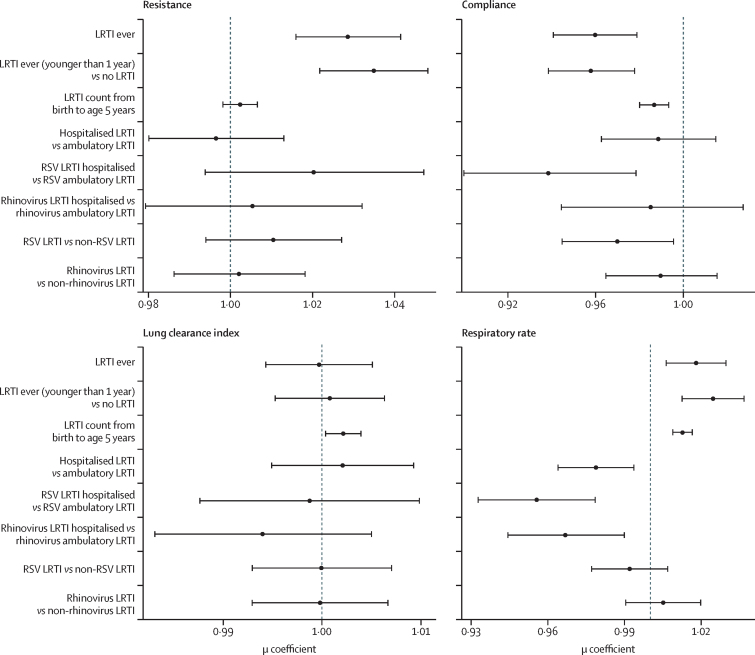
Association of specified LRTIs with the development of lung function parameters from birth to 5 years Each row indicates the adjusted estimates from separate models for each LRTI stratification that included age (months), adjustment for the lung function step change at age 3 years, and the full set of predictors. Higher resistance, lung clearance index, higher respiratory rate, and lower compliance indicate potential impairment in lung function. LTRI=lower respiratory tract infection. RSV=respiratory syncytia

Compared with children who never had a LRTI, those with a LRTI had reduced FRC and compliance and higher resistance and respiratory rate up to age 5 years, independent of risk conferred by baseline lung function (Table 2, Table 3). These finding are consistent with impaired lung growth. Association of baseline lung function with subsequent LRTI is shown in the appendix (p 22) and has been previously described in this cohort.^21^ We further investigated the effect of LRTI frequency, timing, hospitalisation, and causation on lung function (figure 2, appendix pp 23–26). Every additional episode of LRTI further increased respiratory rate (1·012 [95% CI 1·009–1·016]) and LCI (1·002 [1·001–1·004]) and reduced tidal volume (0·996 [0·993–0·998]) and compliance (0·987 [0·98–0·99]). If LRTI occurred before age 1 year, there was an additional reduction in tidal volume (0·98 [0·97–0·99]) and increase in respiratory rate (1·03 [1·01–1·04]). The effect of hospitalised and ambulatory LRTI was similar; however, this differed for RSV, as RSV LRTI requiring hospitalisation was associated with impaired lung function development compared with an ambulatory RSV LRTI, with lower compliance (0·94 [0·90–0·98]) up to age 5 years. RSV LRTI compared with non-RSV LRTI was associated with a lower t_PTEF_/t_E_ (0·97 [0·95–0·99]), consistent with airflow obstruction. Rhinovirus LRTI compared with non-rhinovirus LRTI was not associated with lower lung function in this cohort.

**Figure 3:**
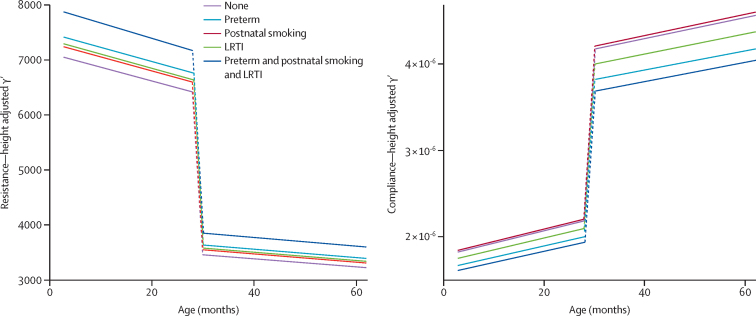
Predicted height-adjusted resistance and compliance by exposure groups over time LRTI=lower respiratory tract infection.

The impact of multiple exposures over 5 years is shown graphically for subgroups of interest (figure 3, appendix pp 19–20). Multiple harmful exposures appear to have an additive effect (figure 3).

The robustness of results was tested by fitting models for the subset of children who had two or more measurements (appendix pp 27–28).

## Discussion

We comprehensively assessed various measures of lung function from birth to age 5 years in South African children, describing changes in lung growth during infancy and the preschool years, and determined the effect of multiple exposures during this key early period. Known factors that affect lung function, such as maternal smoking and asthma, were found to negatively affect lung development, as were other risk factors including HIV exposure and living in poor households. Early-life LRTI impaired lung growth and development, with recurrent LRTI associated with additional impairment for each LRTI. RSV, the leading cause of childhood LRTI globally, was an important cause of impairment in lung development, particularly in children hospitalised with RSV LRTI. Children with a previous RSV LRTI had lower expiratory flow ratio compared with those with non-RSV LRTI. By contrast, rhinovirus LRTI had a similar effect to non-rhinovirus LRTI.

In this birth cohort, we previously reported that early-life LRTI independently affected lung function to age 2 years.^12^, ^13^ In the current study, we extend this finding to show that the effect of early-life LRTI, even an ambulatory event, persists and increases to age 5 years. This finding is of clinical importance, as lung function when children first enter school is strongly predictive of the physiological peak reached in early adulthood.^25^ Our data are consistent with studies from HICs showing that LRTI in childhood is associated with lower spirometric lung function in later life.^6^, ^9^ However, we have now shown that in South Africa, the effect of RSV on lung development is substantial and larger than the effect of rhinovirus, which is the virus most strongly associated with childhood obstructive airway disease in HICs. We have shown that RSV LRTI requiring hospitalisation had a greater effect on lung development than ambulatory RSV LRTI; however, this pattern was not observed for all-cause or rhinovirus LRTI. Further, in comparison to non-RSV LRTI, children with RSV LRTI had a reduced t_PTEF_/t_E_ trajectory, in keeping with development of airway obstruction as a consequence of early-life RSV LRTI.^8^, ^18^ This study highlights the importance of RSV prevention, particularly in high burden settings such as South Africa, and the need to prioritise careful follow-up of children hospitalised with RSV LRTI.

We provide novel evidence that in addition to causative organism and severity, the recurrence and timing of early-life LRTI worsen its effect on lung development. Recurrent events were associated with additional adverse effects on lung volumes and an increase in LCI, suggesting early peripheral airway damage. If LRTI occurred in the first year of life, the sustained effect was larger than if occurring later, suggesting that lung growth effects are strongest with early harm.

Wheezing phenotypes were previously derived in this cohort.^18^ The extensive lung function measures were differentially associated with wheeze phenotypes, providing some insights into the mechanics behind them. Late-onset wheeze was associated with an increase in respiratory resistance over time, consistent with developing reduced airway calibre. Recurrent wheeze through childhood was associated with higher end expiratory resistance and lower compliance, expiratory flow ratio, and respiratory rate throughout pre-school years, consistent with persistent airflow limitation.

Being born very preterm (<32 weeks gestational age) has well documented lifelong implications on respiratory health.^26^ In addition, moderate to late preterm birth (32 to <37 weeks) might affect long-term respiratory health.^27^ Most (69·3%) children born preterm in our cohort were moderate to late preterm.^26^ Preterm-born children had decreased lung function over time, independent of size (both at birth and postnatally) and other risk factors, such as intercurrent LRTI. This association between preterm birth and lung function is particularly important given the global burden of preterm birth and the effect this could have on long-term morbidity and mortality.

HIV exposure increases the risk for LRTI in infancy and alters lung function at birth.^23^ We have previously shown impairments in LCI and resistance at age 2 years in children HEU whose mothers had severe HIV disease.^15^ The current study extends this finding by showing that in children HEU, lung function development is impaired from birth to age 5 years in this high-risk group, with increases in LCI showing a sustained effect of HIV exposure throughout childhood in children whose mothers had a detectable HIV viral load during pregnancy.

Postnatal maternal smoking was associated with altered development of lung function consistent with airway obstruction. This effect was additional to the impact of antenatal smoke exposure. Prenatal maternal psychological adversity, including maternal intimate partner violence, has been associated with increased risk of childhood wheeze and early-life LRTI,^28^ and, as previously described in this cohort, impaired lung function in infancy.^16^ These effects could be explained through direct effects of hypothalamic–pituitary–adrenal axis dysregulation increasing corticotropin-releasing hormone, cortisol, and catecholamines, high concentrations of which can compromise immunological and lung development. Additionally, indirect effects of poorer maternal dietary and lifestyle habits during pregnancy could contribute to impaired child lung growth.^16^, ^29^ We have shown in this analysis the effect of postnatal maternal IPV on lung function development, including impaired expiratory flow ratio t_PTEF_/t_E_, up to the age of 5 years, which is consistent with our previously observed increased risk of wheeze.^18^ This finding highlights the importance of antenatal, perinatal, and postnatal care in optimising lifelong health.

We have also shown that multiple exposures appear additive, with children with co-existing risk factors having the poorest lung development. This finding is important in risk stratification and prioritising preventive interventions and management planning.

The development of lung function parameters differed between male and female children. Sex differences in lung function are well described and reflect larger size-adjusted airway to lung volumes in male children during infancy and differing patterns of airway-parenchyma–somatic growth relationships throughout childhood.^30^

Most children in this cohort were from poor households, with 35·3% living in extreme poverty (household income <US$67 per month). Early-life social disadvantage is associated with lower lung function and its long-term health consequences.^31^ We showed that increasing household income (even to a modest amount) and improved access to maternal education was associated with improved lung development in eary life, although the multiple confounding factors are appreciated. Effects of social disadvantage are probably mediated by a high prevalence of additional risk factors for respiratory disease in the poorest households. In addition, agency, choice, and access to healthy decisions are restricted by poverty, with lifelong consequences for health.

Strengths of this study include the very high cohort retention with detailed longitudinal collection of exposure data and strong surveillance for LRTI and environmental factors. The comprehensive lung function measures throughout early childhood are unique, especially in an LMIC context such as South Africa. The measures fill a knowledge gap in the literature on lung function across the life course and provide detailed assessment of lung growth and function over time, giving insight into mechanisms of developmental impairment. Limitations of this study include that adequately adjusting for the timing of multiple exposures is challenging in an observational cohort. In addition, multiple exposures and comparisons might lead to spurious findings; however, we adjusted our analysis for multiple comparisons and the associations remained. Rhinovirus C is the subtype most strongly associated with airway obstruction; a limitation of our data is that we did not subtype rhinovirus isolates.

This study assessed lung development during infancy and the preschool years and the effect of multiple exposures on this development, identifying factors that impair development and reduce lung function up to age 5 years. Given the importance of lung function achieved in childhood on all-cause mortality in later life, these are important findings that have implications for health across the life course. We have not yet assessed the association of the impaired lung development with subsequent risk for chronic respiratory disease, nor investigated subgroups of children who catch-up or worsen over time; this is important future research that we plan on doing.

In conclusion, multiple early-life factors affected healthy lung growth and development during early childhood, several of which are amenable to public health intervention. Children with multiple risk factors are particularly at risk for impaired lung development. Strengthening interventions to prevent LRTI, such as maternal and childhood vaccination, is key. New RSV preventive interventions for infants make prevention of RSV LRTI a possibility, but access and affordability in LMICs must be assured.

## Data sharing

An anonymised, de-identified version of the dataset can be made available on request to allow all results to be reproduced. All requests should be directed to HJZ, the Drakenstein Child Health Study Principal Investigator.

## Declaration of interests

AC reports personal fees from Stallergenes Greer, AstraZeneca, GlaxoSmithKline, and Worg Pharmaceuticals, outside the submitted work. All other authors declare no competing interests.
