# Supplementary material for: Determinants of lung function development from birth to age 5 years: an interrupted time series analysis of a South African birth cohort
Source: Lancet Child Adolesc Health. 2024 Jun;8(6):400–12. doi: 10.1016/S2352-4642(24)00072-5 (PMC11096865; doi:10.1016/S2352-4642(24)00072-5)
Supplement: Supplementary appendix [file mmc1.pdf]

THE LANCET
Child & Adolescent Health

Supplementary appendix

This appendix formed part of the original submission and has been peer reviewed. We post it as supplied by the authors.

Supplement to: McCready C, Zar HJ, Chaya S, et al. Determinants of lung function development from birth to age 5 years: an interrupted time series analysis of a South African birth cohort. *Lancet Child Adolesc Health* 2024; published online April 12. https://doi.org/10.1016/S2352-4642(24)00072-5.

Determinants of lung function development from birth through school age in a South African birth cohort

Carlyle McCready MSc[1,2], Prof Heather J Zar PhD[2], Shaakira Chaya MD[2], Carvern Jacobs MSc[2], Lesley Workman MPH[2], Prof Zoltan Hantos PhD[3], Prof Graham L Hall PhD[4], Prof Peter D Sly PhD[5], Prof Mark P Nicol PhD[6] ,Prof Dan J Stein PhD[7], Anhar Ullah PhD[8], Prof Adnan Custovic PhD[8], Prof Francesca Little PhD[1], Diane M Gray PhD[2]

[1]Department of Statistical Sciences University of Cape Town, South Africa; [2]Department of Paediatrics and Child Health, and SA-MRC Unit on Child and Adolescent Health, University of Cape Town, South Africa; [3]Department of Anaesthesiology and Intensive Therapy, Semmelweis University, Budapest, Hungary; [4]Children’s Lung Health, Telethon Kids Institute, School of Allied Health, Curtin University, Perth, Australia; [5]Children's Health and Environment Program, Child Health Research Centre, The University of Queensland, Australia; [6]Marshall Centre, School of Biomedical Sciences, University of Western Australia, Perth, Australia; [6]Dept of Psychiatry & Mental Health, and SA-MRC Unit on Risk & Resilience, University of Cape Town, South Africa; [7]National Heart and Lung Institute, Imperial College London, UK

Supplementary appendix

Table of contents

Abbreviations 3
Supplementary methods 3
    Drakenstein Child Health Study (DCHS) 3
    Definition of variables 4
    Lung function testing 6
Statistical analysis 7
    Height-adjusted lung function 7
Supplementary results 10

Abbreviations

BH: Benjamini and Hochberg
CI: Confidence Interval
COPD: Chronic Obstructive Pulmonary Disease
DCHS: Drakenstein Child Health Study
FRC: Functional Residual Capacity
GAIC: Generalised Akaike Information Criterion
GAMLSS: Generalised Additive Models for Location, Scale and Shape
HICs: High-Income Countries
HEU: HIV Exposed Uninfected
HIV: Human Immunodeficiency Virus
IPV: Intimate Partner Violence
IQR: Interquartile Range
ITS: Interrupted Time Series
LCI: Lung Clearance Index
LMIC: Low and Middle-Income Country
LRTI: Lower Respiratory Tract Infection
MBW: Multiple Breath Washout
MRC: Medical Research Council
NIH: National Institutes of Health
Osc: Oscillometry
PCR: Polymerase Chain Reaction
Ree: End-Expiratory Resistance
Rrs: Respiratory System Resistance
RR: Respiratory Rate
RSV: Respiratory Syncytial Virus
RV: Rhinovirus
SES: Socioeconomic Status
TBFVL: Tidal Breathing Flow Volume Loops
tPTEF/tE: Ratio of time to reach peak tidal expiratory flow to total expiratory time
USD: United States Dollar
VT: Tidal Volume
Xee: End-Expiratory Reactance
Xrs: End-Expirator Reactance
Zrs: Respiratory Impedance

SUPPLEMENTARY METHODS

Drakenstein Child Health Study (DCHS)

Pregnant women were recruited at antenatal clinics at two public health facilities in a peri-urban area in South Africa between 5 March 2012 to 31 March 2015 during their second trimester of pregnancy.(1) Inclusion criteria were 18 years or older, 20-28 weeks gestation of pregnancy, and resident in the area. All births occurred at the single public hospital, where birth parameters were obtained by study staff. The study was approved by the Faculty of Health Sciences Human Research Ethics Committee, University of Cape Town and Western Cape Provincial Research committee.

Mother-child pairs were followed from birth with study visits synchronised with immunization visits (diphtheria, tetanus, acellular pertussis, *H. influenzae* b and inactivated polio vaccine at 6, 10, 14 weeks and 18 months, measles vaccine at 9 and 18 months and 13-valent PCV at 6 weeks, 14 weeks and 9 months). Additional study visits were done 2-weekly in the first year in an intensive subset, and thereafter 6-monthly through 5 years in all.

Follow-up and cohort retention were optimized through community workers, a dedicated study phone line available to all participants at all times and intensive face-to-face follow-up of the cohort. Disenrollment followed at least 3 unsuccessful attempts (phone calls and home visits) by study staff to locate participants.

Definition of variables

Current wheeze: Wheezing was assessed using ISAAC questionnaires or was diagnosed on auscultation by trained study staff at a study visit or during an intercurrent illness.(3) Current wheeze was defined as a positive response to the question "Has your child had wheezing or whistling in the chest in the last 12 months?" at each follow-up.

Early-life risk factors: Data on risk factors for wheezing from the antenatal period through 5 years were collected, including sociodemographic factors, nutrition, maternal physical and mental health, home environment, birth factors and breastfeeding(1), Table S1. Maternal mental health measures included measurements of depression, psychological distress, and intimate partner violence (IPV) antenatally and postnatally.(2) Smoking was assessed by maternal self-report antenatally and postnatally. Socioeconomic status (SES) was assessed through a validated measure comprising 4 components: household income, asset ownership, household size and maternal education.(1)

Lower Respiratory Tract Infection (LRTI): Active surveillance was used to confirm LRTI(3,4); all episodes were assessed by trained study staff and defined by WHO case definitions as:

(1) episode of LRTI (cough or difficulty breathing and increased respiratory rate or lower chest wall in-drawing in a child aged >2 months); or

(2) severe LRTI (child aged <2 months with increased respiratory rate or lower chest wall in-drawing, or any general danger sign in a child of any age).

At each LRTI or wheezing episode, a nasopharyngeal swab (FLOQSwabsTM, Copan Diagnostics, CA) was obtained. Nucleic acid was extracted using mechanical lysis on a Tissuelyzer LT (Qiagen, Germany) followed by extraction with the QIAsymphony® Virus/Bacteria mini kit (Qiagen, Germany). Quantitative multiplex real-time PCR (qPCR) was done using FTDResp33 (Fast-track Diagnostics, Luxembourg), identifying up to 33 organisms including respiratory syncytial virus (RSV), rhinovirus (RV) and adenovirus (AV).(4)

Table S1: Definition of variables

Variable	Measurement used
**Child characteristics**	
**Birth weight z-score**	Z-scores were calculated using the Fenton newborn growth charts for preterm infants and WHO growth charts for term-born infants. Z-scores adjust for age and sex, with the use of Fenton and WHO in combination to adjust for prematurity. Trained clinical staff recorded newborn birthweight at the time of delivery, using a digital scale with a precision level of 10 g.
**Preterm**	Gestational age at birth <37 weeks. Gestational age was estimated using antenatal ultrasound measurements in the second trimester; if this was unavailable then symphysis-fundal height or maternal recall if the last menstrual period was used
**Late preterm**	Gestational age at birth 34 to <37 weeks.
**Lower Respiratory Tract Infection (LRTI)**	World Health Organization (WHO) criteria were used to define LRTI.(1) LRTI was diagnosed in children with a cough or difficulty breathing and age-specific tachypnoea (≥50 breaths per minute for children aged 2–12 months; > 40 for children 1-5 year) or if the child had lower chest wall indrawing. Severe LRTI was diagnosed in children younger than 2 months with tachypnoea (>60 breaths per min) or lower chest wall indrawing, or in children of any age if the child had a general danger sign (cyanosis, unable to drink, seizures, or decreased level of consciousness). Episodes of LRTI less than 28 days apart were regarded as a single episode.
**RSV-LRTI, RV-LRTI**	A LRTI episode with a positive PCR result for RSV or RV on a nasopharyngeal swab.
**Wheeze phenotypes**	Never, Early transient, Late onset, and Recurrent wheeze, as previously described.(2)
**HIV exposed uninfected**	A child born to a mother living with HIV but who was uninfected. HIV-exposed children were tested for HIV at 6 weeks (by PCR), 9 months (by PCR, ELISA or rapid antibody testing) and 18 months (by rapid antibody testing), as per provincial prevention of mother-to-child transmission guidelines
**Maternal characteristics**	
**Smoking**	Self-reported smoking was assessed using the Alcohol, Smoking and Substance Involvement Screening Test (ASSIST) and self-report antenatally, at birth and during the past three months postnatally. Postnatal smoking was defined at any time point from birth to 12 Months (3, 4)
**Maternal asthma or allergy**	Self-reported maternal asthma or allergy was assessed by direct interview antenatally.
**Intimate partner violence (IPV)**	IPV Questionnaire adapted from the WHO multi-country study was used to assess maternal physical, emotional, or sexual violence exposure antenatally and postnatally. Postnatal IPV collection was used from birth to 1-year. This included the 10 week, 6 month, and 12 month data points. (3)
**Depression**	The Edinburgh Postnatal Depression Scale (EPDS) was used to measure maternal depression antenatally and postnatally. 10 Questions were scored 0-3 and totalled. A cut-off value of 13 was used to separate the participants into above- or below-threshold groups. Postnatal depression was used from birth to 1-year. This included the 10 week, 6 month, and 12 month data points. (3)
**Socio-economic status (SES)**	
**Income**	Average household income per month at maternal enrolment. Categories are Less than R1 000 (USD67), R1 000 (USD67) to R5 000 (USD336), and more than R5 000 (USD336).
**Asset ownership**	Asset ownership is a summed score of 13 different questions including access to electricity, tap or running water, domestic servant, flush toilet inside, built-in kitchen sink, an electric stove or hotplate, working telephone, at least one motor car or truck, motorcycle or scooter, a bicycle, shop at supermarkets, use any financial services, account at a retail store. The distribution across quartiles at maternal enrolment. The levels are Low, Low-Medium, Medium-High, and High.
**Household size**	The distribution across quartiles of household size (members) at maternal enrolment. The levels are: Small (1-3), Small-Medium (4), Medium-Large (5-6), Large (7-18)

Maternal education	Highest maternal education level obtained at maternal enrolment at maternal enrolment. The levels are primary; some secondary; completed secondary education; and any tertiary education.

RSV = respiratory syncytial virus.; RV = Rhinovirus, HIV = Human immunodeficiency virus

Lung function testing

The measures until 2 years were collected during behaviourally assessed quiet sleep, with the infant lying supine, head in the neutral position and breathing through a face mask and filter. Measurements were made in accordance with current recommended standards for collection and analysis (5, 6) and as previously published.(7) Oscillometry to measure respiratory impedance (Zrs) was collected using the custom-made wave-tube setup (University of Szeged, Hungary).(8, 9) Conventional measurement of Zrs spectra using a pseudo-random signal between 8 and 48 Hz was used. From at least three reproducible 30-s Zrs recordings, mean values of respiratory impedance were obtained. Conventional oscillometry measures included mean respiratory system resistance (R) and compliance (C) which were determined from model fitting to the measured Zrs data in the frequency range 12-32 Hz for R and 8-32 Hz for C.(8, 9) This procedure is illustrated in Figure S1, with data from preschool measurements (see below). From the 16-Hz intra-breath measurements end-expiratory resistance (R_{ee}) and reactance (X_{ee}) were determined.

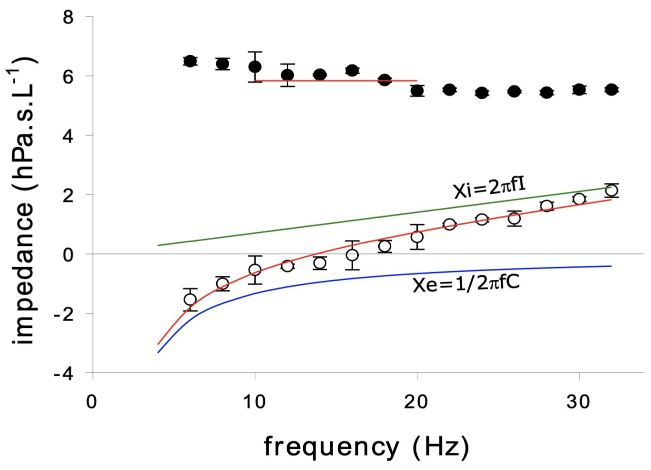

Figure S1: Illustration of the resistance (R) - inertance (I) – compliance (C) model fitting to measured preschool impedance data. Mean values of resistance (•) and reactance (◦) from repeated measurements, whiskers indicate standard deviation. Model fitting curves are plotted in red. R was obtained as the mean value in the 10-20-Hz frequency (f) range; I and C were obtained from fitting the reactance (X) data by the model $X=2\pi fI-1/(2\pi fC)$. Green and blue lines, respectively, illustrate the inertial (Xi) and elastic (Xe) components of the total X.

Tidal breathing and multiple breath washout (MBW) measures were collected using the Exhalyzer D with ultrasonic flow meter (Ecomedics AG, Duernten, Switzerland). The infant MBW measures were performed using 4·0% sulphur hexafluoride as a tracer gas and with acquisition and analysis software (Wbreath version 3·28·0; Ndd Medizintechnik), as previously described.(10, 11)

The preschool measures (ages 3 to 5 years) were collected with children awake and sitting. Oscillometry measures were collected with a custom-made system (INCIRCLE wave-tube, University of Szeged, Hungary).(12) Children performed quiet tidal breathing through a mouthpiece and filter, with nose-clip and cheeks firmly supported. Conventional Zrs measures were collected using a pseudo-random signal 6-32 Hz. Measurements consisted of a maximum of five 16-s epochs of multifrequency oscillations to yield a minimum of three acceptable measurements, i.e. without any vocal cord noise, apnoea, irregular breathing pattern, glottic closure, leak or sighs. R and C were estimated in the frequency range 10-20 Hz and 6-26 Hz, respectively.(12) R_{ee} and X_{ee} were determined

from 10-Hz intra-breath recordings. Preschool tidal breathing and MBW measures were collected using the Exhalyzer D with ultrasonic flow meter (Ecomedics AG, Duernten, Switzerland) with a child sitting, quietly breathing through a facemask and filter. The Nitrogen (N_2) washout test was employed, using 100·0% O_2 to washout the endogenous N_2 gas and using analysis software Spiroware 3·3·1, Ecomedics AG, Duernten, Switzerland. Tests were performed with the child breathing comfortably through a size 2 silicone facemask (Laerdal) and filter (Gibeck Humid-Vent Filter). The dead space of the mask was determined by water displacement. Measurements included: tidal breathing flow volume loops (TBFVL) which included tidal volume (VT), respiratory rate (RR) and expiratory flow ratio of time of peak total expiratory flow to time of expiration (t_{PTEF}/t_E), MBW measurements include the functional residual capacity (FRC) and the lung clearance index (LCI). Tests were collected and assessed for quality in accordance with recent recommendations for preschool MBW testing (13) and oscillometry.(14)

Statistical analysis

Height-adjusted lung function

The lung function measures were adjusted for height following the methods described by Cole et al.(15) Figure S2 below shows the FRC outcome versus height for each annual visit before (Y) and after (Y') height adjustment to remove the relationship between lung function outcome and height.

Figure S2: FRC vs Height for each annual visits before (Y) and after (Y') height adjustment

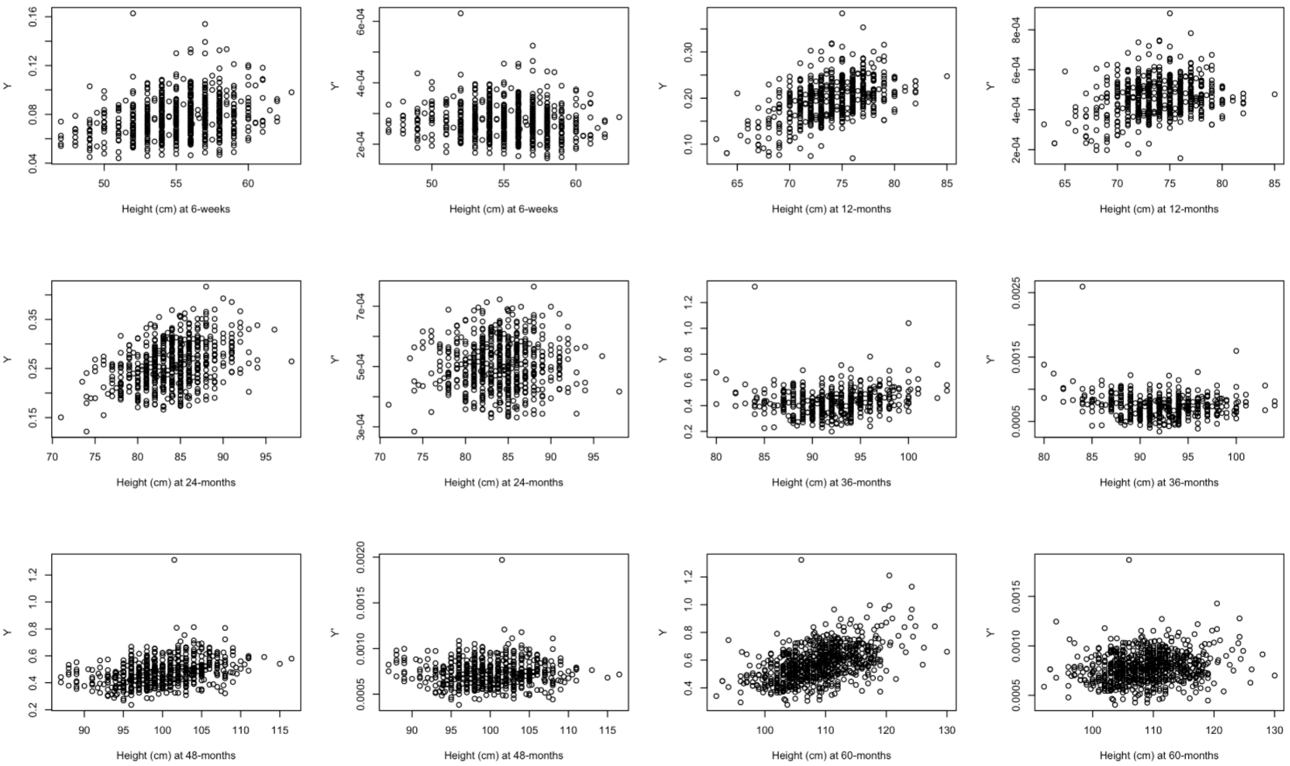

Table S2: Fitted models for each distribution

Model	Distribution	Parameter	Model formulation	
1	BCCG	μ	$\mu = \beta_0 + \beta_1 T + \beta_2 X_t + \beta_3 TX_t$	
		σ	$\sigma = \beta_0 + \beta_1 T + \beta_2 X_t + \beta_3 TX_t$	
		ν	$\nu = \beta_0$	
2	BCCG	μ	$\mu = \beta_0 + \beta_1 T + \beta_2 X_t + \beta_3 TX_t$	*+ predictor*
		σ	$\sigma = \beta_0 + \beta_1 T + \beta_2 X_t + \beta_3 TX_t$	
		ν	$\nu = \beta_0$	
3	BCCG	μ	$\mu = \beta_0 + \beta_1 T + \beta_2 X_t + \beta_3 TX_t$	*+ predictor*
		σ	$\sigma = \beta_0 + \beta_1 T + \beta_2 X_t + \beta_3 TX_t$	
		ν	$\nu = \beta_0 + \beta_1 T$	
4	BCT	μ	$\mu = \beta_0 + \beta_1 T + \beta_2 X_t + \beta_3 TX_t$	
		σ	$\sigma = \beta_0 + \beta_1 T + \beta_2 X_t + \beta_3 TX_t$	
		ν	$\nu = \beta_0$	
		τ	$\tau = \beta_0$	
5	BCT	μ	$\mu = \beta_0 + \beta_1 T + \beta_2 X_t + \beta_3 TX_t$	*+ predictor*
		σ	$\sigma = \beta_0 + \beta_1 T + \beta_2 X_t + \beta_3 TX_t$	
		ν	$\nu = \beta_0$	
		τ	$\tau = \beta_0$	
6	BCT	μ	$\mu = \beta_0 + \beta_1 T + \beta_2 X_t + \beta_3 TX_t$	*+ predictor*
		σ	$\sigma = \beta_0 + \beta_1 T + \beta_2 X_t + \beta_3 TX_t$	
		ν	$\nu = \beta_0 + \beta_1 T$	
		τ	$\tau = \beta_0 + \beta_1 T$	

All models include a child-specific random effect.
T: age (months)
X_t: a dummy variable indicating the different time periods (< 3 years or > 3 years)
BCCG: Box-Cox Cole and Green distribution
BCT: Box-Cox t distribution

Figure S3: Directed acyclic graph (DAG) for evaluation of exposures.

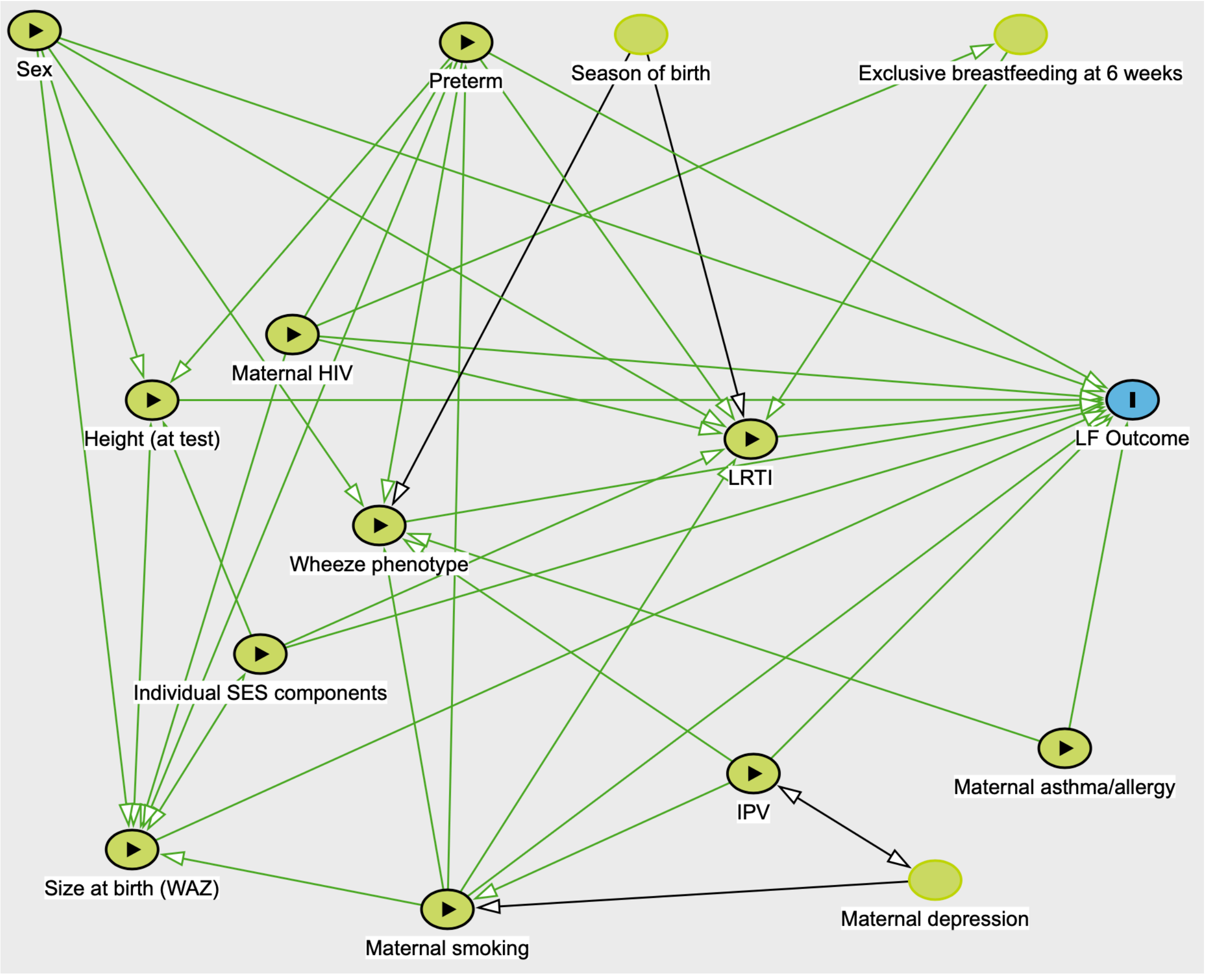

For each lung function outcome, we investigated a model containing the DAG identified set: sex, prematurity, wheeze phenotypes, previous LRTI, weight-for-age z-score (at birth), SES components, maternal smoking, maternal HIV, maternal asthma or allergy, and intimate partner violence (IPV). All models additionally contained *age*, a constant (X_t) representing the size of the step-change at 3 years, an *age*: X_t interaction term, and a child-specific random effect.

SUPPLEMENTARY RESULTS

The average length of follow-up of children was 64·3 (SD 4·6) months, representing 4830 child-years of follow-up.

Table S3: Comparison of characteristics of included versus excluded children.

	Total cohort N=1143	Included (N=966)	No lung function at 5 years of age (N=177)*	P-value
**Infant characteristics**				
Sex (male)	589/1143 (51·5%)	494/966 (51·3%)	95/177 (53·7%)	0·59
Pre-term (<37 weeks)	192/1143 (16·8%)	150/966 (15·5%)	42/177 (23·7%)	0·0011
HIV exposed uninfected	248/1143 (21·7%)	211/966 (21·8%)	37/177 (20·9%)	0·86
Median weight-for-age z-score (waz) at birth [IQR]	-0·25 [-0·92, 0·42]	-0·25 [-0·96, 0·41]	-0·23 [-0·76, 0·49]	0·59
**Maternal characteristics**				
Median age [IQR] at enrolment (years)	25·76 [21·99, 30·82]	26·13 [22·08, 30·84]	24·15 [21·51, 29·79]	0·0034
Antenatal smoking	324/1143 (28·3%)	290/966 (30·0%)	34/177 (19·2%)	0·0044
Maternal allergy	80/1143 (7·0%)	66/966 (6·8%)	14/177 (7·9%)	0·72
**Socio economic status (SES)**				
Low	286/1143 (25·0%)	242/966 (25·0%)	44/177 (24·9%)	0·99
Low-moderate	286/1143 (25·0%)	252/966 (26·0%)	34/177 (19·2%)	0·064
Moderate-high	286/1143 (25·0%)	248/966 (25·7%)	38/177 (21·5%)	0·27
High	285/1143 (24·9%)	224/966 (23·2%)	61/177 (34·5%)	0·0019

*Excluded includes 162 children not followed to 5 years and 15 without successful lung function testing
IPV = intimate partner violence, IQR = interquartile range, HIV = Human immunodeficiency virus

Table S4: Summary of lung function outcomes at each visit.

	Number	Percentage[1]	Mean (sd)
Functional residual capacity (litres)			
6-weeks	734	76·0%	0·077 (0·016)
1-year	593	61·4%	0·20 (0·043)
2-years	599	62·0%	0·26 (0·045)
3-years	449	46·5%	0·43 (0·90)
4-years	557	57·7%	0·48 (0·096)
5-years[2]	702	72·7%	0·59 (0·13)
Lung clearance index (number of FRC turnovers)			
6-weeks	734	76·0%	7·15 (0·44)
1-year	593	61·4%	6·77 (0·56)
2-years	599	62·0%	6·69 (0·47)
3-years	449	46·5%	7·00 (0·77)
4-years	557	57·7%	6·93 (0·59)
5-years[2]	702	72·7%	6·84 (0·56)
Resistance ($hPa.s.L^{-1}$)			
6-weeks	677	70·1%	47·59 (14·89)
2-years	568	58·8%	26·19 (10·25)
3-years	425	44·0%	11·48 (2·21)
4-years	614	63·6%	10·13 (2·13)
5-years[2]	750	77·6%	8·76 (1·93)
Compliance ($L.hPa^{-1}$)			
6-weeks	677	70·1%	0·001 (0·001)
2-years	568	58·8%	0·002 (0·001)
3-years	425	44·0%	0·005 (0·002)
4-years	614	63·6%	0·006 (0·002)
5-years[2]	750	77·6%	0·007 (0·002)
t_{PTEF}/t_E (%)			
6-weeks	781	80·9%	37·96 (12·07)
1-year	630	65·2%	29·39 (10·09)
2-years	621	64·3%	27·28 (8·91)

3-years	494	51·1%	42·84 (11·82)
4-years	668	69·2%	42·18 (12·13)
5-years[2]	777	80·4%	38·92 (10·93)
Tidal volume (millilitres)			
6-weeks	781	80·9%	34·80 (6·34)
1-year	630	65·2%	92·95 (14·22)
2-years	621	64·3%	119·46 (16·59)
3-years	506	52·4%	178·56 (28·97)
4-years	668	69·2%	202·27 (33·77)
5-years[2]	777	80·4%	229·43 (49·38)
Respiratory rate (breaths per minute)			
6-weeks	781	80·9%	48·83 (11·29)
1-year	630	65·2%	29·51 (5·04)
2-years	621	64·3%	26·33 (4·32)
3-years	499	51·7%	28·01 (5·72)
4-years	667	69·1%	26·66 (6·19)
5-years[2]	777	80·4%	27·73 (8·00)
Ree (hPa.s.L^{-1})			
6-weeks	498	51·6%	45·46 (12·59)
2-years	567	58·7%	19·89 (6·96)
3-years	394	40·8%	12·32 (2·77)
4-years	580	60·0%	10·59 (2·54)
5-years[2]	694	71·8%	8·93 (2·36)
Xee (hPa.s.L^{-1})			
6-weeks	498	51·6%	-8·21 (8·00)
2-years	567	58·7%	-0·76 (3·31)
3-years	394	40·8%	-2·63 (1·71)
4-years	580	60·0%	-2·08 (1·53)
5-years[2]	694	71·8%	-1·46 (1·24)

[1]A denominator of 966 active children was used.
t_{PTEF}/t_E = Ratio time of peak total expiratory flow to time of expiration; R_{ee} = respiratory resistance at the end of expiration; X_{ee} = respiratory reactance at the end of expiration; sd=standard deviation.
[2]Age at 5-year in months: median 61.96, min 59.03, max 68.96

Figure S4: Longitudinal plots of measured and size adjusted (on right) lung function outcomes from 6 weeks and annually to 5 years.

(a) Functional residual capacity, (b) lung clearance index, (c) respiratory rate, (d) tidal volume, (e) time to peak tidal expiratory flow over total expiratory time (t_{PTEF}/t_E), (f) resistance, (g) compliance, (h) respiratory resistance at the end of expiration (Ree), and (i) respiratory reactance at the end of expiration (Xee) versus age stratified by sex. (Note: Figure 2 (i), right) illustrates the height-adjusted Xee after inverting and adding a constant term. The dotted line between 2 years and 3 years highlights the step change between testing methods. The height adjustment changes the scale of the lung function outcomes, while the overall shape of development from 0-5 years is mostly similar between the original and height-adjusted outcomes.

(a)

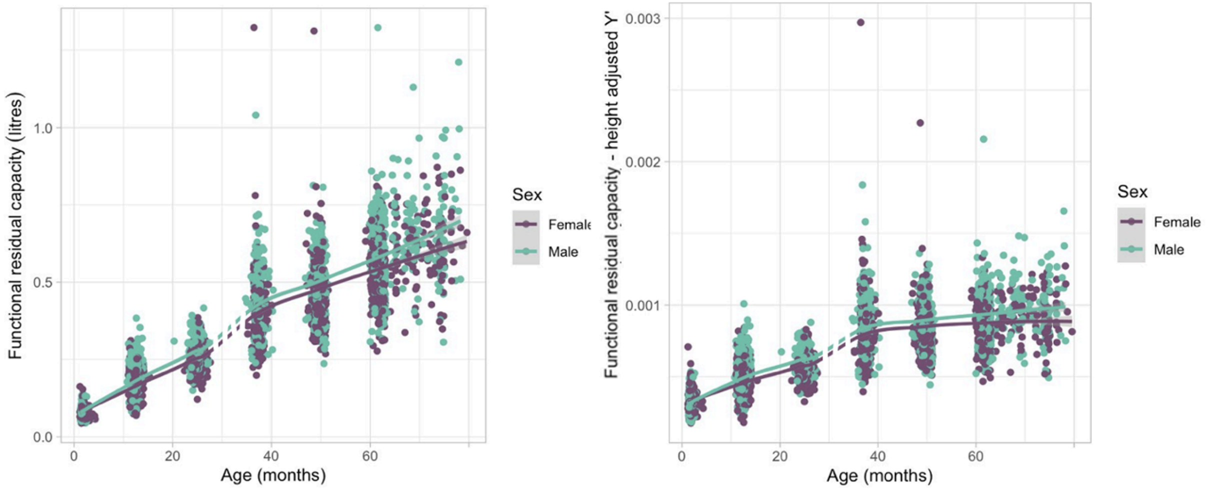

(b)

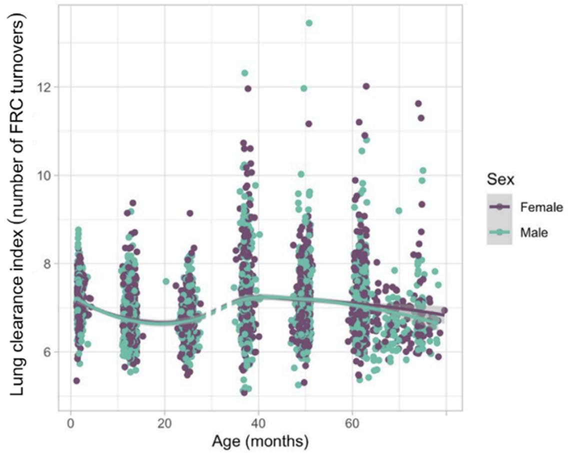

(c)

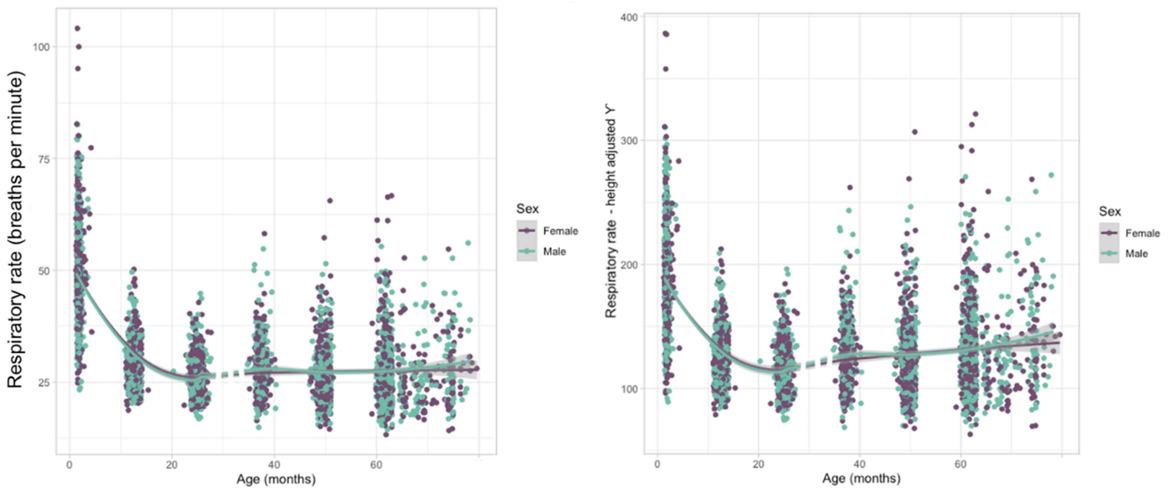

Respiratory rate (breaths per minute)
Age (months)
Sex
Female
Male
Respiratory rate - height adjusted Y'
Age (months)
Sex
Female
Male

(d)

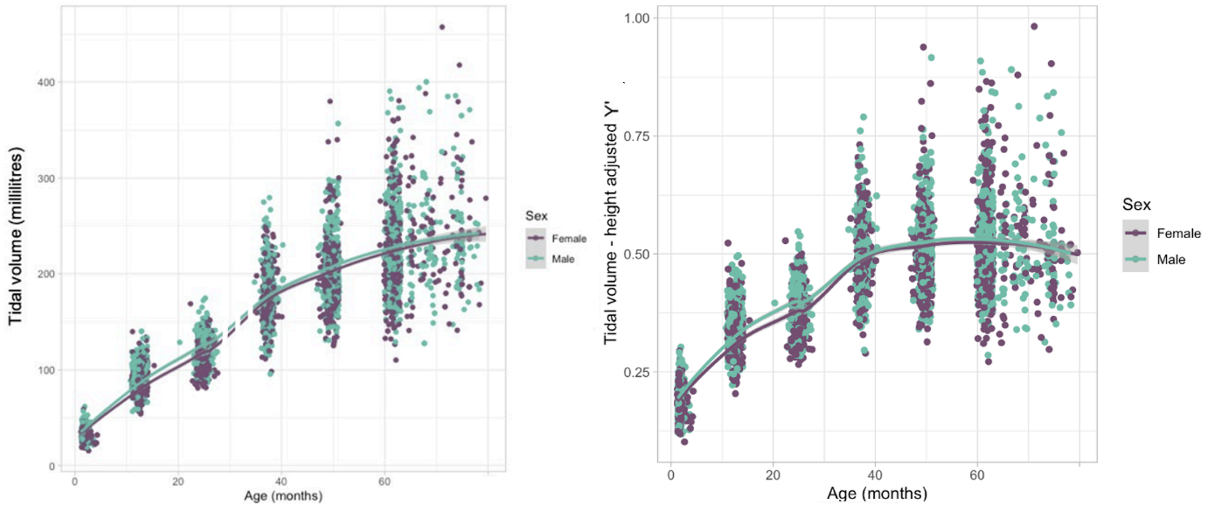

Tidal volume (millilitres)
Age (months)
Sex
Female
Male
Tidal volume - height adjusted Y'
Age (months)
Sex
Female
Male

(e)

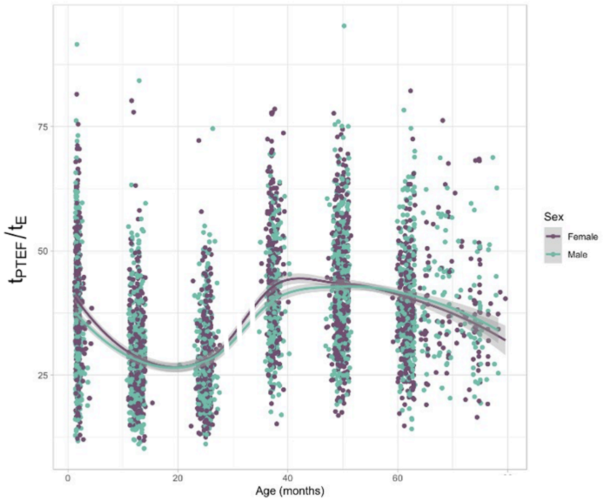

tPTEF/tE
Age (months)
Sex
Female
Male

(f)

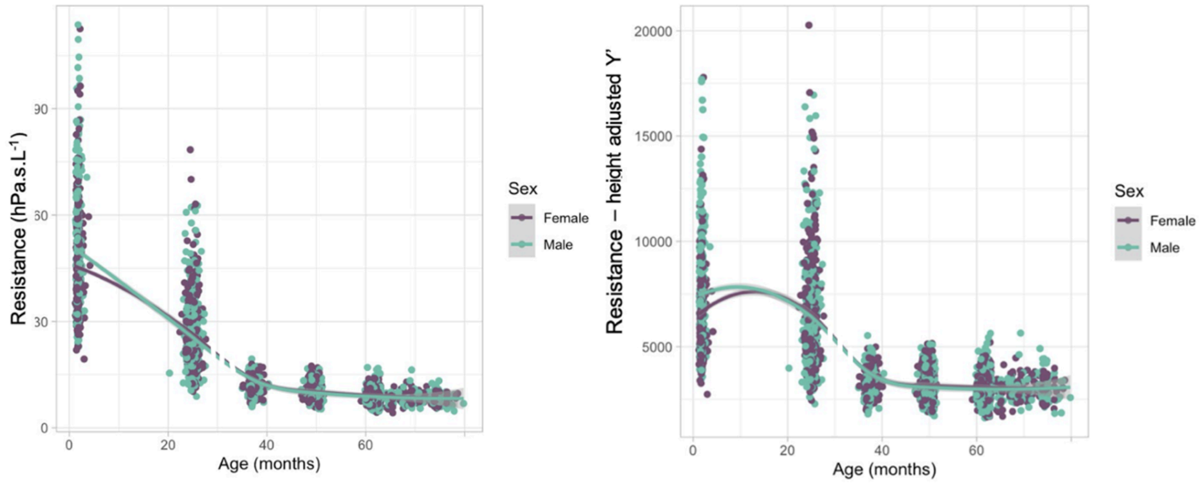

Resistance (hPa.s.L-1)
60
30
0
0
20
40
60
Age (months)
Sex
Female
Male
Resistance – height adjusted Y'
20000
15000
10000
5000
0
20
40
60
Age (months)
Sex
Female
Male

(g)

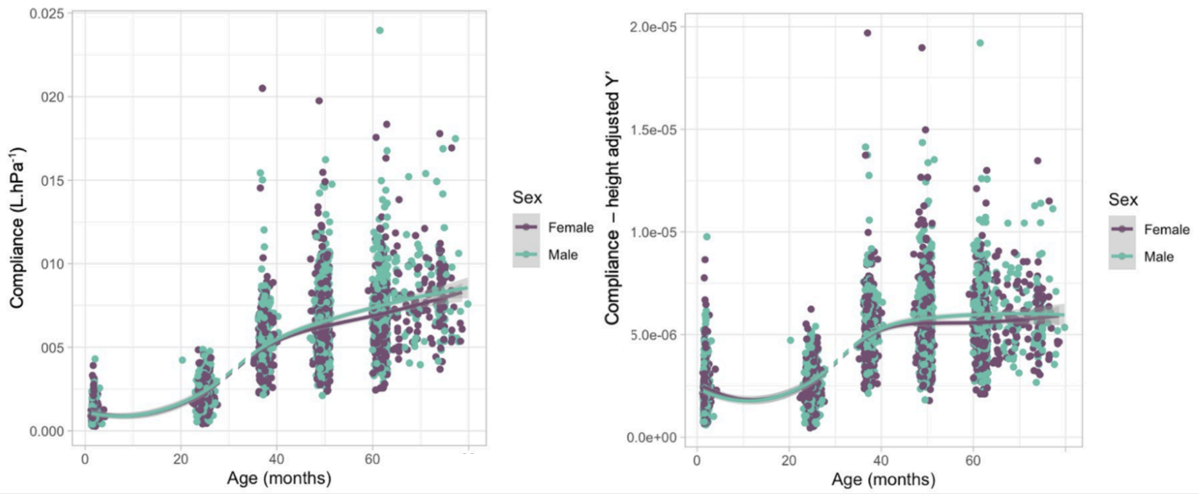

Compliance (L.hPa-1)
0.025
020
015
010
005
0.000
0
20
40
60
Age (months)
Sex
Female
Male
Compliance – height adjusted Y'
2.0e-05
1.5e-05
1.0e-05
5.0e-06
0.0e+00
0
20
40
60
Age (months)
Sex
Female
Male

(h)

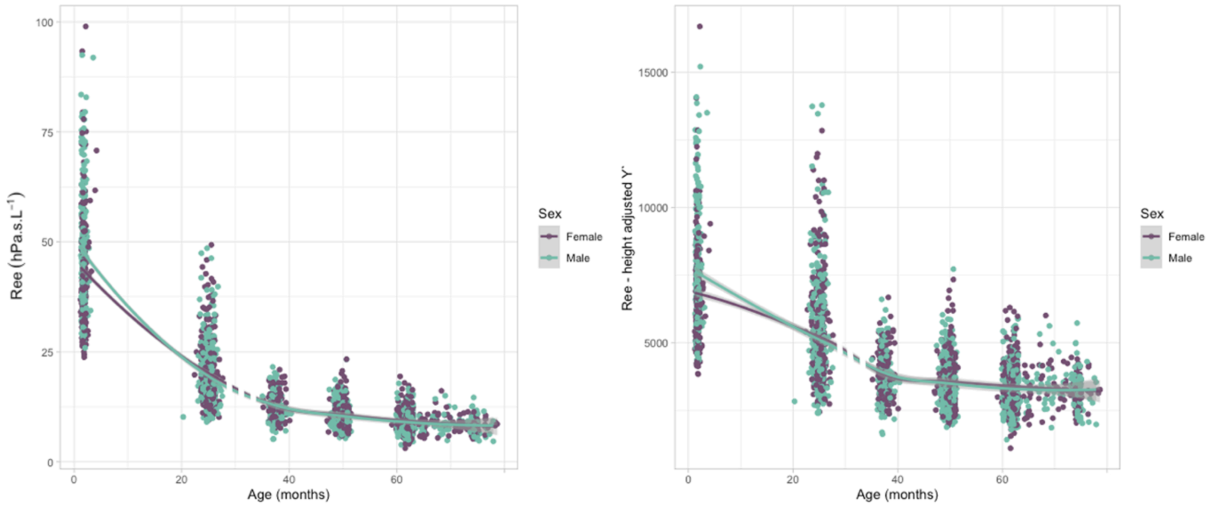

Ree (hPa.s.L-1)
100
75
50
25
0
0
20
40
60
Age (months)
Sex
Female
Male
Ree - height adjusted Y'
15000
10000
5000
0
20
40
60
Age (months)
Sex
Female
Male

(i)

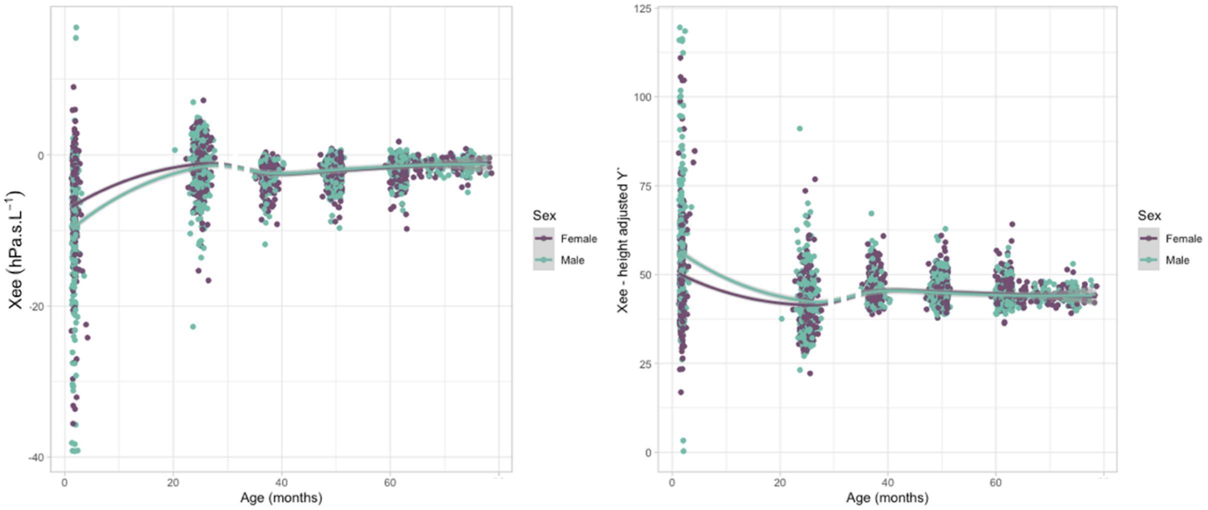

Xee (hPa.s.L⁻¹)
Age (months)
Sex
Female
Male
Xee - height adjusted Y`
Age (months)
Sex
Female
Male

Figure S5: Lung function quartiles determined at 6-weeks and then tracked over the first 5 years of childhood.

Mean (a) functional residual capacity, (b) lung clearance index, (c) respiratory rate, (d) tidal volume, (e) time to peak tidal expiratory flow over total expiratory time (t_{PTEF}/t_E), (f) resistance, (g) compliance, (h) respiratory resistance at the end of expiration (Ree), and (i) respiratory reactance at the end of expiration (Xee) versus age stratified by starting quartile.

(a)

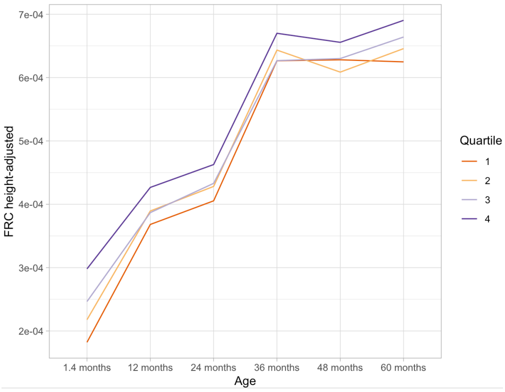

(b)

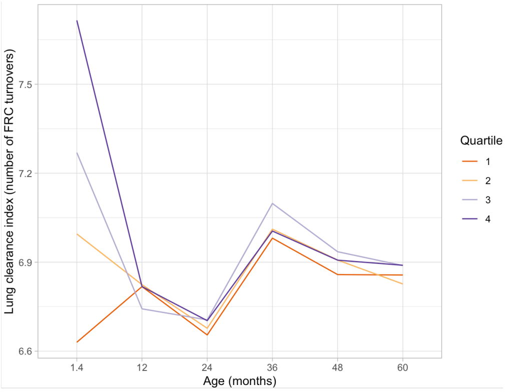

(c)

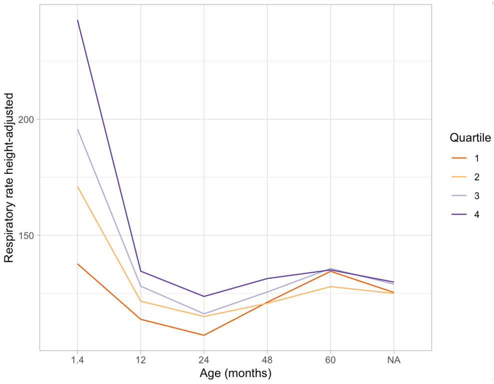

(d)

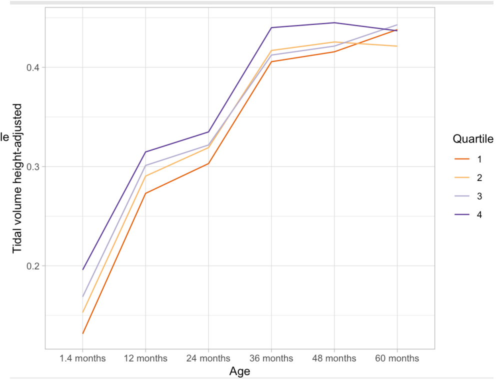

(e)

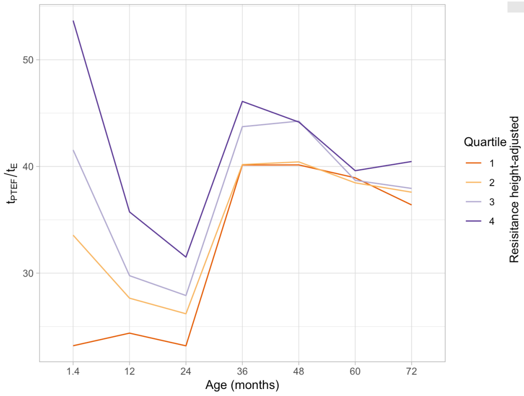

(f)

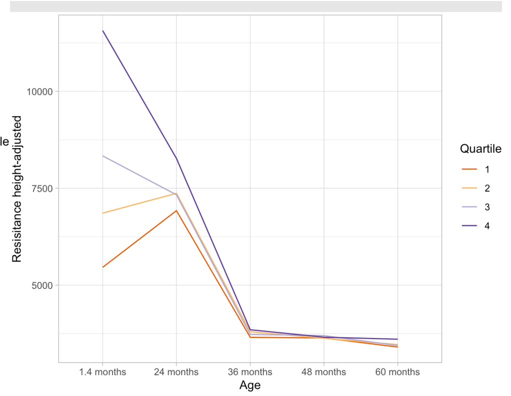

(g)

(h)

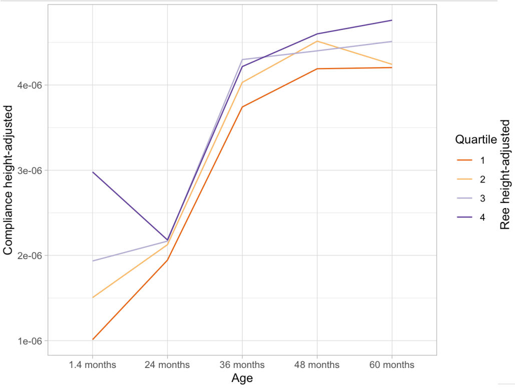
Compliance height-adjusted
4e-06
3e-06
2e-06
1e-06
1.4 months
24 months
36 months
48 months
60 months
Age
Quartile
1
2
3
4

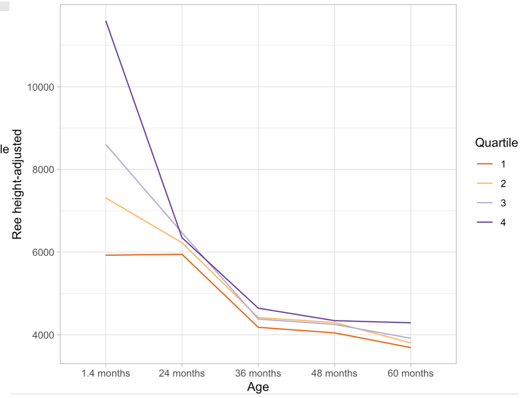
Ree height-adjusted
10000
8000
6000
4000
1.4 months
24 months
36 months
48 months
60 months
Age
Quartile
1
2
3
4

(i)

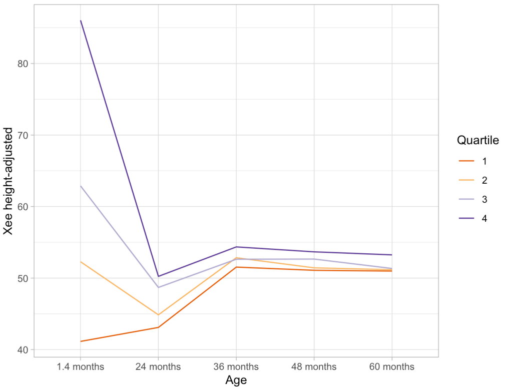
Xee height-adjusted
80
70
60
50
40
1.4 months
24 months
36 months
48 months
60 months
Age
Quartile
1
2
3
4

Figure S6: Predicted height-adjusted resistance and compliance subgroups over age (a) with and without LRTI, (b) with and without LRTI in children, stratified by sex, (c) with and without LRTI in children, stratified by preterm, (d) with and without LRTI in children, stratified by HEU, and (e) with and without LRTI in children, stratified by smoke exposure.

(a)

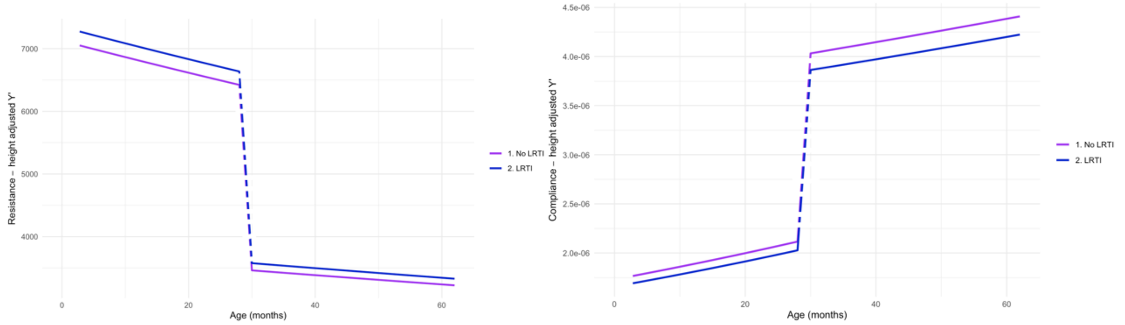

(b)

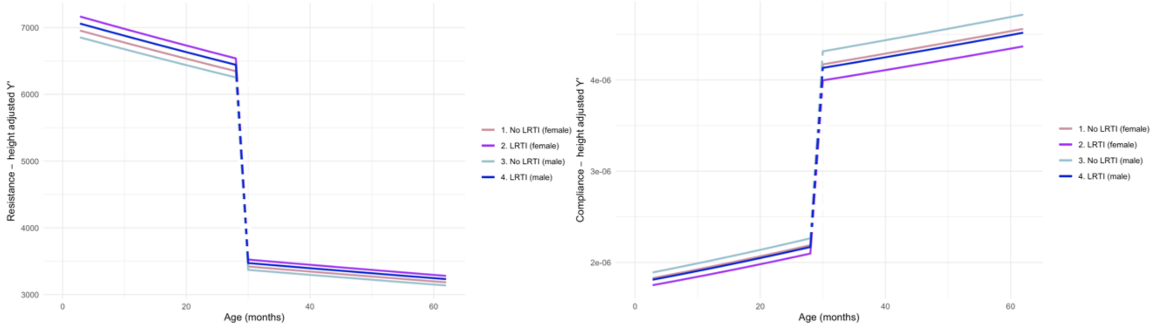

(c)

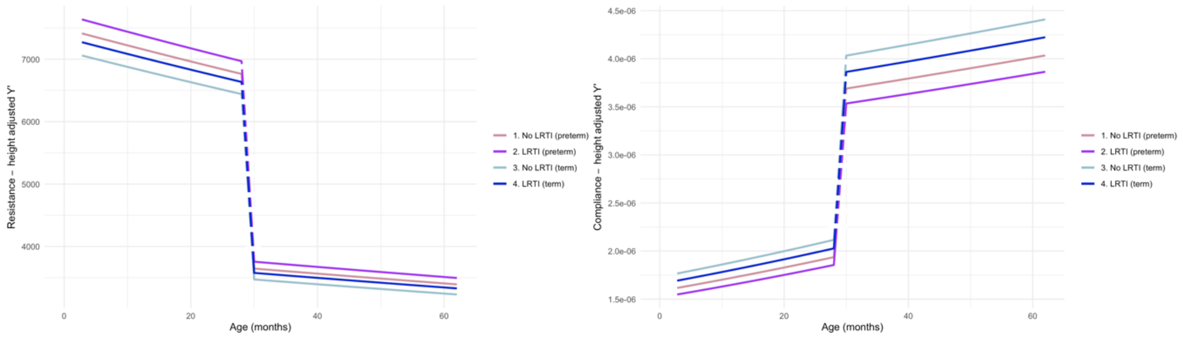

(d)

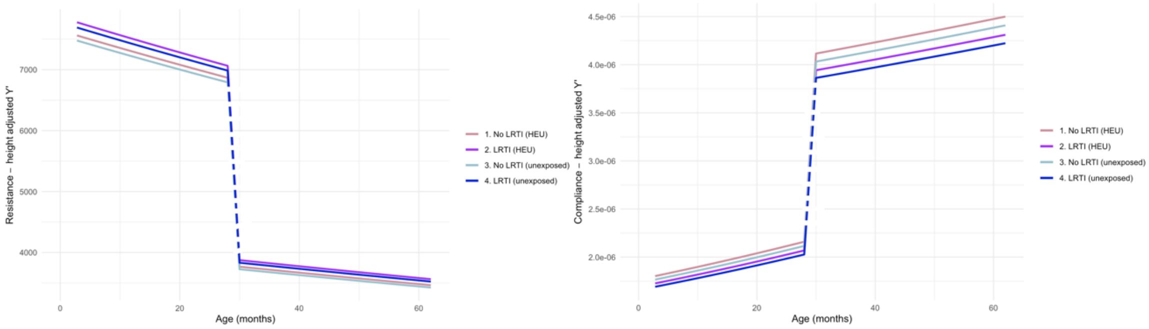
Resistance – height adjusted Y'
7000
6000
5000
4000
0
20
40
60
Age (months)
1. No LRTI (HEU)
2. LRTI (HEU)
3. No LRTI (unexposed)
4. LRTI (unexposed)
Compliance – height adjusted Y'
4.5e-06
4.0e-06
3.5e-06
3.0e-06
2.5e-06
2.0e-06
0
20
40
60
Age (months)
1. No LRTI (HEU)
2. LRTI (HEU)
3. No LRTI (unexposed)
4. LRTI (unexposed)

(e)

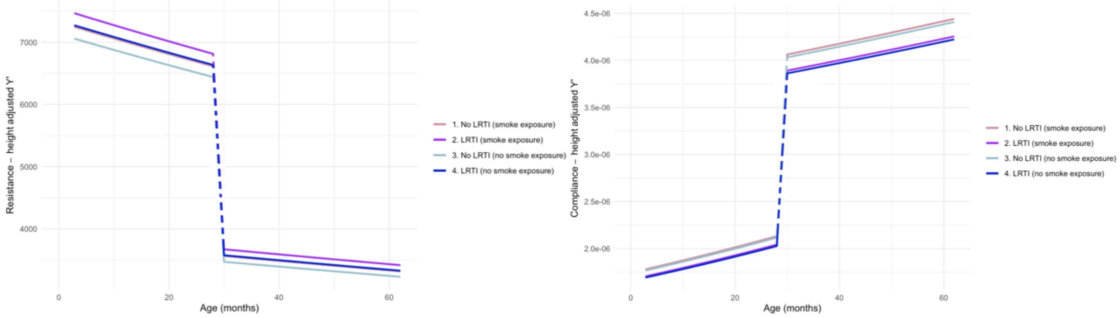
Resistance – height adjusted Y'
7000
6000
5000
4000
0
20
40
60
Age (months)
1. No LRTI (smoke exposure)
2. LRTI (smoke exposure)
3. No LRTI (no smoke exposure)
4. LRTI (no smoke exposure)
Compliance – height adjusted Y'
4.5e-06
4.0e-06
3.5e-06
3.0e-06
2.5e-06
2.0e-06
0
20
40
60
Age (months)
1. No LRTI (smoke exposure)
2. LRTI (smoke exposure)
3. No LRTI (no smoke exposure)
4. LRTI (no smoke exposure)

Table S5: Association of maternal HIV disease severity with lung clearance index, tidal volume, and respiratory system reactance at the end of expiration (Xee) from birth to 5 years.

	LCI[1]		Tidal volume[1]		Inversed Xee[2]	
	Effect size (95% CI)	P-value	Effect size (95% CI)	P-value	Effect size (95% CI)	P-value
HIV exposed uninfected versus HIV unexposed	1.008 (1.002-1.014)	0·016	1.022 (1.011-1.034)	p<0·0001	1.004 (0.998-1.009)	0·12
Maternal Cd4 categories during pregnancy						
(>= 500 cells/ mm3) versus maternal HIV negative	1.006 (0.996-1.016)	0·21	1·038 (1·021-1·056)	p<0·0001	1·011 (1·003-1·018)	0·0046
(350-500 cells/ mm3) versus maternal HIV negative	1.005 (0.993-1.017)	0·41	1·006 (0·984-1·029)	0·61	1·008 (0·997-1·019)	0·14
(< 350 cells/ mm3) versus maternal HIV negative	1.009 (0.999-1.019)	0·062	1·011 (0·993-1·029)	0·25	1·011 (1·003-1·019)	0·0059
Maternal Viral load (vl) categories during pregnancy						
(Lower than detectable limit) versus maternal HIV negative	1.006 (0.997-1.014)	0·21	1·011 (0·996-1·027)	0·16	1·017 (1·01-1·025)	p<0·0001
(VL detectable >=40-1000 copies/ml) versus maternal HIV negative	1.017 (1.004-1.031)	0·013	1·01 (0·986-1·036)	0·41	1·02 (1·009-1·031)	p<0·0001
(Virally unsuppressed >1000 copies/ml) versus maternal HIV negative	1.022 (1.006-1.038)	0·008	1·036 (1·006-1·067)	0·020	1·011 (0·997-1·026)	0·12

The first three estimates for cd4 categories and the last three estimates for viral load categories are from separate models that included age (months) and 3 year testing step change adjustment.
Xee = respiratory reactance at the end of expiration; LCI = lung clearance index; HIV = human immunodeficiency virus
[1] Box-Cox-t (BCT) distribution
[2] Box-Cox Cole and Green (BCCG) distribution
Effect size: exponent of the mu-coefficients

Table S6: Differences in baseline (6 week) lung function between children who develop LRTI and those that did not have a LRTI.

	Height-adjusted lung function outcomes		
	Never had LRTI ever [median (IQR)]	*Had LRTI ever [median (IQR)]*	*P-value*[1]
**Functional residual capacity**	0·00027 (0·00024-0·00031)	0·000265 (0·00023-0·00031)	0·064
**Lung clearance index (n turnovers)***	7·09 (6·87-7·42)	7·13 (6·88-7·43)	0·51
**Tidal volume**	0·19 (0·17-0·21)	0·18 (0·16-0·20)	0·33
**Respiratory rate**	178·11 (154·35-203·93)	189·51 (162·27-217·20)	p<0·001
**t_{PTEF}/t_E**	39·5 (30·3-46·8)	35·6 (28·3-44·8)	0·0023
**Compliance**	0·0022 (0·0017-0·0028)	0·0020 (0·0015-0·0026)	0·050
**Resistance**	6502·52 (5469·26-8036·63)	6763·83 (5534·95-8314·71)	0·11
**Inverted Xee**	55·11 (47·50-65·59)	61·89 (51·71-75·40)	0·0012
**Ree**	8308·11 (7141·18-9873·54)	8694·81 (7420·76-10459·66)	0·21

[1]Mood's Median Test was used to compare medians. *Height adjustments are not required for Lung clearance index (n turnovers) and t_{PTEF}/t_E; t_{PTEF}/t_E = time to peak tidal expiratory time over total expiratory time; Ree = respiratory resistance at the end of expiration; Xee = respiratory reactance at the end of expiration.

Table S7: Association of specific LRTI with the development of multiple breath washout and tidal breathing parameters from birth to 5 years

	FRC[1]		LCI[1]		Respiratory rate[1]		Tidal volume[2]		t_{PTEF}/t_E:[1]	
	Effect size (95% CI)	P-value (adjusted for multiple comparisons[3)])	Effect size (95% CI)	P-value (adjusted for multiple comparisons[3)])	Effect size (95% CI)	P-value (adjusted for multiple comparisons[3)])	Effect size (95% CI)	P-value (adjusted for multiple comparisons[3)])	Effect size (95% CI)	P-value (adjusted for multiple comparisons[3)])
LRTI ever	0.987 (0.975-0.998)	0·022 (0·055)	0.999 (0.994-1.005)	0·91 (0·97)	1.018 (1.0063-1.029)	0·0024 (0.0067)	0.993 (0.984-1.003)	0·18 (0.31)	1.002 (0.984-1.020)	0·79 (0.82)
LRTI ever under 1 year (versus no LRTI)	0.990 (0.979-1.002)	0·11 (0·20)	1.001 (0.995-1.006)	0·79 (0.94)	1.025 (1.013-1.037)	p<0·0001 (p<0·0001)	0.986 (0.977-0.996)	0·0059 (0.013)	1.005 (0.987-1.023)	0·59 (0.69)
LRTI count birth to 5 years	0.997 (0.9931-1.001)	0·12 (0·21)	1.002 (1.0003-1.004)	0·019 (0·095)	1.012 (1.009-1.016)	p<0·0001 (p<0·0001)	0.996 (0.993-0.998)	0·0081 (0.018)	0.999 (0.994-1.006)	0·92 (0.92)
Hospitalised LRTI (vs ambulatory LRTI)	1.010 (0.995-1.026)	0·21 (0·29)	1.002 (0.995-1.009)	0·58 (0·85)	0.979 (0.964-0.994)	0·0054 (0·015)	0.997 (0.984-1.010)	0·64 (0.73)	1.001 (0.977-1.024)	0·95 (0.95)
RSV-hospitalised LRTI (vs RSV-ambulatory LRTI) –	0.989 (0.966-1.013)	0·39 (0·51)	0.998 (0.988-1.010)	0·82 (0·95)	0.955 (0.933-0.978)	p<0·0001 (p<0·0001)	1.003 (0.983-1.023)	0·76 (0.86)	0.985 (0.949-1.023)	0·43 (0.54)
RV-hospitalised LRTI (vs RV-ambulatory LRTI)	1.008 (0.984-1.032)	0·51 (0·64)	0.994 (0.983-1.005)	0·28 (0·72)	0.967 (0.944-0.989)	0·0051 (0·014)	1.019 (0.999-1.039)	0·062 (0.14)	1.001 (0.965-1.039)	0·95 (0.95)
RSV-LRTI (vs non-RSV LRTI)	1.014 (0.998-1.029)	0·076 (0·15)	0.999 (0.993-1.007)	0·98 (0·98)	0.992 (0.977-1.007)	0·29 (0·40)	1.013 (0.999-1.0252	0·051 (0.012)	0.971 (0.949-0.994)	0·014 (0.032)

RV-LRTI (vs non-RV LRTI)	1.008 (0.993-1.022)	0·31 (0·43)	0.999 (0.993-1.007)	0·95 (0·97)	1.005 (0.991-1.020)	0·49 (0·56)	1.004 (0.992-1.016)	0·51 (0.58)	1.018 (0.995-1.041)	0·13 (0·22)

Each row indicates the adjusted estimates from separate models for each LRTI stratification that included age (months), a dummy variable indicating the different time periods (< 3 years or > 3 years), and the full set of predictors as shown in Table 2: sex (male), preterm (<37 weeks), HIV exposed uninfected versus HIV unexposed, birthweight z-score, wheeze phenotype, LRTI ever, postnatal IPV, maternal asthma/allergy, postnatal smoking, maternal education, asset ownership, household size, and income.

All models include a child-specific random effect.

[1] Box-Cox-t (BCT) distribution

[2] Box-Cox Cole and Green (BCCG) distribution

[3] An approach by Benjamini, Y., and Hochberg, Y. (1995) for multiple testing.

FRC = functional residual capacity; LCI = lung clearance index; t_{PTEF}/t_E = time to peak tidal expiratory time over total expiratory time; IPV = intimate partner violence, LRTI = lower respiratory tract infection, RSV = respiratory syncytial virus.; RV = Rhinovirus

The models for LCI and t_{PTEF}/t_E: were done using the unstandardised measurements.

Effect size: exponent of the mu-coefficients

Table S8: Association of LRTIs with the development of oscillometry parameters from birth to age 5 years.

	Resistance[1]		Compliance[1]		Ree[1]		Inversed Xee[2]	
	Effect size (95% CI)	P-value (adjusted for multiple comparisons[3])	Effect size (95% CI)	P-value (adjusted for multiple comparisons[3])	Effect size (95% CI)	P-value (adjusted for multiple comparisons[3])	Effect size (95% CI)	P-value (adjusted for multiple comparisons[3])
LRTI ever	1.028 (1.016-1.041)	p<0·0001 (p<0·0001)	0.959 (0.941-0.978)	p<0·0001 (p<0·0001)	1.034 (1.017-1.051)	p<0·0001 (p<0·0001)	1.0059 (1.0015-1.0104)	0·0083 (0·029)
LRTI ever under 1 year (versus no LRTI)	1.035 (1.022-1.048)	p<0·0001 (p<0·0001)	0.958 (0.938-0.978)	p<0·0001 (p<0·0001)	1.037 (1.020-1.054)	p<0·00001 (p<0·001)	1.011 (1.006-1.015)	p<0·0001 (p<0·0001)
LRTI count birth to 5 years	1.002 (0.998-1.006)	0·26 (0·54)	0.987 (0.980-0.993)	p<0·0001 (p<0·0001)	1.007 (1.002-1.013)	0·0079 (0·025)	1.003 (1.001-1.004)	p<0·0001 (p<0·0001)
Hospitalised LRTI (vs ambulatory LRTI)	0.996 (0.980-1.013)	0·67 (0·79)	0.988 (0.963-1.015)	0·39 (0·46)	1.001 (0.980-1.022)	0.91 (0·92)	1.007 (1.001-1.013)	0·021 (0·052)
RSV-LRTI hospitalised (VS RSV-ambulatory LRTI)	1.020 (0.994-1.047)	0·13 (0·29)	0.938 (0.899-0.978)	0·0030 (0·0080)	1.022 (0.988-1.057)	0·21 (0·33)	1.009 (1.0001-1.019)	0·048 (0·10)
RV-LRTI hospitalised (VS RV-ambulatory LRTI)	1.005 (0.979-1.032)	0·69 (0·82)	0.985 (0.944-1.027)	0·48 (0·54)	1.006 (0.973-1.041)	0·71 (0·89)	1.002 (0.993-1.012)	0·64 (0·84)
RSV-LRTI (vs non-RSV LRTI)	1.011 (0.994-1.027)	0·21 (0·43)	0.969 (0.945-0.995)	0·022 (0·055)	1.017 (0.995-1.038)	0·12 (0·23)	1.014 (1.008-1.019)	p<0·0001 (p<0·0001)
RV-LRTI (vs non-RV LRTI)	1.002 (0.986-1.018)	0·79 (0·86)	0.989 (0.965-1.015)	0·43 (0·11)	1.013 (0.992-1.033)	0·23 (0·36)	1.003 (0.997-1.009)	0·28 (0·43)

Each row indicates the adjusted estimates from separate models for each LRTI stratification that included age (months), a dummy variable indicating the different time periods (< 3 years or > 3 years), and the full set of predictors as shown in Table 2: sex (male), preterm (<37 weeks), HIV exposed uninfected versus HIV unexposed, birthweight z-score, wheeze phenotype, LRTI ever, postnatal IPV, maternal asthma/allergy, postnatal smoking, maternal education, asset ownership, household size, and income.

All models include a child-specific random effect.

[1] Box-Cox-t (BCT) distribution

[2] Box-Cox Cole and Green (BCCG) distribution

[3] An approach by Benjamini, Y., and Hochberg, Y. (1995) for multiple testing.

Ree = respiratory resistance at the end of expiration; Xee = respiratory reactance at the end of expiration; IPV = intimate partner violence, LRTI = lower respiratory tract infection, RSV = respiratory syncytial virus; RV = Rhinovirus

Effect size: exponent of the mu-coefficients

Table S9: Association of early life risk factors with the development of functional residual capacity (FRC), tidal volume (VT), and resistance from birth to age 5 years excluding while excluding subject with less than 2 measurements; multivariate model

	FRC[1]		Tidal volume[2]		Resistance[1]	
	**Effect size (95% CI)**	**P-value**	**Effect size (95% CI)**	**P-value**	**Effect size (95% CI)**	**P-value**
Age (month) for 0-to- 3-years	1.023 (1.022-1.024)	p<0·0001	1.025 (1.024-1.026)	p<0·0001	0.996 (0.994-0.997)	p<0·0001
Adjustment to LF measurement at age 3	2.117 (2.047-2.188)	p<0·0001	2.316 (2.254-2.381)	p<0·0001	0.532 (0.514-0.552)	p<0·0001
Adjustment to 0-to- 3-year age effect beyond age 3	0.980 (0.979-0.981)	p<0·0001	0.976 (0.975-0.977)	p<0·0001	1.0012 (0.999-1.0028)	0·12
**Child characteristics**						
Sex (male)	1.062 (1.051-1.073)	p<0·0001	1.031 (1.023-1.040)	p<0.0001	0.978 (0.967-0.989)	p<0·0001
Preterm (<37 weeks)	0.982 (0.968-0.9965)	0·014	0.976 (0.965-0.988)	p<0.0001	1.058 (1.042-1.075)	p<0·0001
HIV exposed uninfected versus HIV unexposed	1.0007 (0.987-1.014)	0·92	1.022 (1.011-1.034)	p<0.0001	1.010 (0.995-1.025)	0·16
Birthweight-z-score	1.0059 (1.001-1.011)	0·019	1.013 (1.0089-1.017)	p<0.0001	0.993 (0.988-0.998)	0·011
**Wheeze phenotype**						
Early transient (vs Never)	0.994 (0.981-1.0079)	0·41	0.992 (0.981-1.0033)	0·17	0.993 (0.979-1.0073)	0·34
Late onset (vs Never)	1.016 (0.998-1.034)	0·061	0.993 (0.979-1.0077)	0·36	1.024 (1.0052-1.044)	0·0097
Recurrent (vs Never)	1.012 (0.996-1.029)	0·15	1.0056 (0.992-1.019)	0·41	1.0089 (0.991-1.026)	0·31
**LRTI**						
LRTI ever	0.987 (0.976-0.998)	0·023	0.994 (0.984-1.0034)	0·20	1.028 (1.016-1.041)	p<0·0001
**Maternal characteristics**						
Postnatal IPV	1.015 (1.0044-1.026)	0·0049	0.999 (0.991-1.0086)	0·96	0.991 (0.979-1.0025)	0·12
Maternal asthma/allergy	1.016 (0.996-1.036)	0·11	0.986 (0.970-1.0023)	0·099	0.994 (0.973-1.016)	0·60

Postnatal smoking	0.985 (0.974-0.996)	0·0095	1.0085 (0.999-1.018)	0·080	1.028 (1.016-1.041)	p<0·0001
**Socio-economic status**						
Maternal education						
Secondary (vs Primary)	0.986 (0.967-1.0056)	0·16	1.012 (0.995-1.028)	0·15	1.010 (0.988-1.032)	0·33
Completed secondary (vs Primary)	0.994 (0.973-1.016)	0·63	1.0067 (0.989-1.025)	0·45	1.026 (1.0044-1.049)	0·022
Any tertiary (vs Primary)	0.979 (0.951-1.0084)	0·15	1.052 (1.028-1.077)	p<0·0001	0.997 (0.966-1.028)	0·84
Asset ownership						
Low-Medium (vs Low)	1.0034 (0.988-1.018)	0·65	1.0012 (0.988-1.014)	0·84	1.0009 (0.985-1.017)	0·91
Medium-High (vs Low)	1.0006 (0.985-1.016)	0·94	1.0049 (0.992-1.018)	0·45	0.998 (0.981-1.015)	0·81
High (vs Low)	1.018 (1.0023-1.034)	0·033	1.022 (1.0081-1.037)	0·0015	1.0055 (0.987-1.024)	0·55
Household size						
Small-Medium (vs small)	0.992 (0.977-1.0078)	0·34	1.0097 (0.997-1.023)	0·14	1.0072 (0.990-1.024)	0·40
Medium-Large (vs small)	1.028 (1.012-1.045)	p<0·0001	1.018 (1.0047-1.032)	0·0073	1.0084 (0.991-1.026)	0·34
Large (vs small)	1.0049 (0.989-1.021)	0·54	0.997 (0.984-1.011)	0·66	0.995 (0.978-1.012)	0·56
Income						
R1 000-5 000 ($67-336) vs (< R1 000 ($67))	1.012 (1.001-1.023)	0·033	1.0034 (0.994-1.013)	0·46	1.0019 (0.990-1.014)	0·75
> R5 000 ($336) vs < R1 000 ($67)	1.021 (1.003-1.039)	0·019	0.993 (0.979-1.0075)	0·35	0.996 (0.977-1.015)	0·68

All models include a child-specific random effect, all variables in the table included in model. All lung function measurements were standardised for height at test.

[1] Box-Cox-t (BCT) distribution

[2] Box-Cox Cole and Green (BCCG) distribution

FRC = functional residual capacity; IPV = intimate partner violence, LRTI = lower respiratory tract infection. Effect size: exponent of the mu-coefficients (percentage change of lung function measure associated with the predictor variable in question compared to the reference category)

References

1. WHO. Integrated management of childhood illness: distance learning course. Geneva: WHO; 2014.
2. McCready C, Haider S, Little F, Nicol MP, Workman L, Gray DM, et al. Early childhood wheezing phenotypes and determinants in a South African birth cohort: longitudinal analysis of the Drakenstein Child Health Study. Lancet Child Adolesc Health. 2022.
3. Stein DJ, Koen N, Donald KA, Adnams CM, Koopowitz S, Lund C, et al. Investigating the psychosocial determinants of child health in Africa: The Drakenstein Child Health Study. Journal of neuroscience methods. 2015;252:27-35.
4. Group WAW. The Alcohol, Smoking and Substance Involvement Screening Test (ASSIST): development, reliability and feasibility. Addiction. 2002;97(9):1183-94.
5. Robinson PD, Latzin P, Verbanck S, Hall GL, Horsley A, Gappa M, et al. Consensus statement for inert gas washout measurement using multiple- and single- breath tests. Eur Respir J. 2013;41(3):507-22.
6. Foong RE, Rosenow T, Simpson SJ, Stoklin B, Gray D, Pillow JJ, et al. End-inspiratory molar mass step correction for analysis of infant multiple breath washout tests. Pediatr Pulmonol. 2017;52(1):10-3.
7. Gray D, Willemse L, Visagie A, Czovek D, Nduru P, Vanker A, et al. Determinants of early-life lung function in African infants. Thorax. 2017;72(5):445-50.
8. Hantos Z, Czovek D, Gyurkovits Z, Szabo H, Maar BA, Radics B, et al. Assessment of respiratory mechanics with forced oscillations in healthy newborns. Pediatr Pulmonol. 2015;50(4):344-52.
9. Gray DM, Czovek D, McMillan L, Turkovic L, Stadler JAM, Vanker A, et al. Intra-breath measures of respiratory mechanics in healthy African infants detect risk of respiratory illness in early life. Eur Respir J. 2019;53(2).
10. Gray D, Willemse L, Visagie A, Smith E, Czövek D, Sly PD, et al. Lung function and exhaled nitric oxide in healthy unsedated African infants. Respirology. 2015;20(7):1108-14.
11. Chaya S, Vanker A, Brittain K, MacGinty R, Jacobs C, Hantos Z, et al. The impact of antenatal and postnatal indoor air pollution or tobacco smoke exposure on lung function at 3 years in an African birth cohort. Respirology. 2023.
12. Sly PD, Hantos Z. The International Collaboration to Improve Respiratory Health in Children (INCIRCLE) ERS Clinical Research Collaboration. European Respiratory Journal. 2018;52(6):1801867.
13. Robinson PD, Latzin P, Ramsey KA, Stanojevic S, Aurora P, Davis SD, et al. Preschool Multiple-Breath Washout Testing. An Official American Thoracic Society Technical Statement. Am J Respir Crit Care Med. 2018;197(5):e1-e19.
14. King GG, Bates J, Berger KI, Calverley P, de Melo PL, Dellaca RL, et al. Technical standards for respiratory oscillometry. Eur Respir J. 2020;55(2).
15. Cole TJ, Stanojevic S, Stocks J, Coates AL, Hankinson JL, Wade AM. Age- and size-related reference ranges: a case study of spirometry through childhood and adulthood. Stat Med. 2009;28(5):880-98.
